# Pseudogene ACTBP2 increases blood–brain barrier permeability by promoting KHDRBS2 transcription through recruitment of KMT2D/WDR5 in Aβ_1–__42_ microenvironment

**DOI:** 10.1038/s41420-021-00531-y

**Published:** 2021-06-14

**Authors:** Qianshuo Liu, Xiaobai Liu, Defeng Zhao, Xuelei Ruan, Rui Su, Xiuli Shang, Di Wang, Chunqing Yang, Yixue Xue

**Affiliations:** 1 Department of Neurobiology, School of life Sciences, China Medical University, Shenyang, China; 2 Key Laboratory of Cell Biology, Ministry of Public Health of China, China Medical University, Shenyang, China; 3 Key Laboratory of Medical Cell Biology, Ministry of Education of China, China Medical University, Shenyang, China; 4 Department of Neurosurgery, Shengjing Hospital of China Medical University, Shenyang, China; 5 Key Laboratory of Neuro-oncology in Liaoning Province, Shenyang, China; 6 The 105th Class, 7-Year Program, China Medical University, Shenyang, China; 7 Department of Neurology, First Affiliated Hospital of China Medical University, Shenyang, China

**Keywords:** Non-coding RNAs, Epigenetics, Blood-brain barrier

## Abstract

The blood–brain barrier (BBB) has a vital role in maintaining the homeostasis of the central nervous system (CNS). Changes in the structure and function of BBB can accelerate Alzheimer’s disease (AD) development. β-Amyloid (Aβ) deposition is the major pathological event of AD. We elucidated the function and possible molecular mechanisms of the effect of pseudogene ACTBP2 on the permeability of BBB in Aβ_1–42_ microenvironment. BBB model treated with Aβ_1–42_ for 48 h were used to simulate Aβ-mediated BBB dysfunction in AD. We proved that pseudogene ACTBP2, RNA-binding protein KHDRBS2, and transcription factor HEY2 are highly expressed in ECs that were obtained in a BBB model in vitro in Aβ_1–42_ microenvironment. In Aβ_1–42_-incubated ECs, ACTBP2 recruits methyltransferases KMT2D and WDR5, binds to KHDRBS2 promoter, and promotes KHDRBS2 transcription. The interaction of KHDRBS2 with the 3′UTR of HEY2 mRNA increases the stability of HEY2 and promotes its expression. HEY2 increases BBB permeability in Aβ_1–42_ microenvironment by transcriptionally inhibiting the expression of ZO-1, occludin, and claudin-5. We confirmed that knocking down of Khdrbs2 or Hey2 increased the expression levels of ZO-1, occludin, and claudin-5 in APP/PS1 mice brain microvessels. ACTBP2/KHDRBS2/HEY2 axis has a crucial role in the regulation of BBB permeability in Aβ_1–42_ microenvironment, which may provide a novel target for the therapy of AD.

## Introduction

Alzheimer’s disease (AD), the most common type of dementia, is a rapidly increasing neurodegenerative disease in the elderly. β-Amyloid (Aβ) deposition is the major pathological event of AD [1, 2]. The blood–brain barrier (BBB) plays a vital role in maintaining the homeostasis of the central nervous system (CNS). BBB is composed of cerebral microvascular endothelial cells (ECs), pericytes, extracellular matrix, and podocytes of perivascular astrocytes [3]. The tight junction between adjacent brain microvascular ECs creates a structural and functional barrier that maintains brain homeostasis [4]. Changes in the structure and function of BBB can cause a cascade of neurotoxicity, neuroinflammation, and oxidative stress, which in turn impacts brain cell function, and accelerates AD development [3]. Evidence from recent studies indicates that Aβ can lead to BBB dysfunction in early stage of AD [5, 6]. The structural and functional changes of BBB precede the changes of glial cells and neurons during the development of AD [7]. Thus, reduction of BBB breakdown may provide a new direction for early treatment of AD.

Pseudogenes are DNA fragments that are highly homologous to the sequence of certain functional genes, however, they do not have protein-coding capability [8]. Some pseudogenes were considered to be biologically similar to lncRNAs owing to the accumulation of mutations in the open-reading frame [9]. Recent researches show that pseudogenes may participate in epigenetic regulation, gene transcription, and post-transcriptional gene regulation, and play an important regulatory role in the development of neurodegenerative diseases, including AD [10]. Actin beta pseudogene 2 ACTBP2 (also known as ACTBP8, SE33), with 1769bp segments, locates in the 5q14.1. ACTBP2 is frequently detected from tumor tissues or in the urine of patients with bladder cancer [11]. There is no research reported about the expression and function of ACTBP2 in neurodegenerative diseases at this moment.

RNA-binding proteins (RBPs) are involved in the post-transcriptional regulatory process, like RNA splicing, transport, stability, translation, and intracellular localization. Studies show that the abnormal structure or expression of RBPs is one of the most common pathological changes in AD, for example, fused in sarcoma (FUS), polyglutamine-binding protein 1 (PQBP1), TAR DNA-binding protein (TDP-43), etc. All these studies recognized the importance of RBPs in AD [12–14]. KH domain-containing, RNA-binding, and signal transduction-associated protein 2 (KHDRBS2) (also known as SLM1), a member of signal transduction and activator of RNA family, is highly expressed in the brain tissue [15, 16]. Genome-wide association studies found negative correlation expression between KHDRBS2 and CRYL1 in AD patients’ temporal cortex and cerebellar neurons. This indicates that KHDRBS2 participates in gene regulation to promote the progress of AD [17]. KHDRBS2 participates in regulating the splicing of a variety of synapse function-related genes, such as Tomosyn2, LysoPLD/ATX, Dgkb, Kif21a, and Cask, and regulates the behavior and cognitive function of AD mice [18].

Lysine (K) methyltransferase 2D (KMT2D, also known as MLL2), a member of SET1 family of methyltransferases, specifically catalyzes the di-methylation and tri-methylation of H3K4 (histone H3 lysine 4 protein) subunit [19]. WD repeat protein 5 (WDR5), as a KMT2D-reactive protein plays an important role in catalyzing the di/tri-methylation of H3K4 [20]. The presence of H3K4me3 is associated with promoters of transcriptionally active genes [21].

HEY2 (hes related family bHLH transcription factor with YRPW motif 2) gene encodes a member of basic/helix-loop-helix (bHLH) proteins. It is an important part of the transcriptional regulatory network, and plays multiple roles in controlling a variety of biological processes, especially the development of the CNS and cardiovascular system [22, 23]. HEY2 participates in maintaining the self-renewal, differentiation of neural stem cells, and regulates the transformation of endocardial epithelial to mesenchymal induced by Notch [24, 25]. The expression of HEY2 increases in the CA1 area of AD mice hippocampus, induces Aβ production, and aggravates cognitive deficits [26].

In this study, we identified the endogenous expression of ACTBP2, KHDRBS2, and HEY2 in ECs of BBB model in Aβ_1–42_ microenvironment. We studied the possibility of ACTBP2 regulating the expression of KHDRBS2 at the epigenetic level by recruiting KMT2D/WDR5 to act on the KHDRBS2 promoter region. Then, we investigated whether KHDRBS2 affected the function of BBB in Aβ_1–42_ microenvironment by changing the stability of HEY2 mRNA. This study provides a new target for AD treatment from the perspective of pseudogene regulating BBB function in the Aβ_1–42_ microenvironment.

## Results

### Pseudogene ACTBP2 increased BBB permeability in Aβ_1–42_ microenvironment

**Fig. 1:**
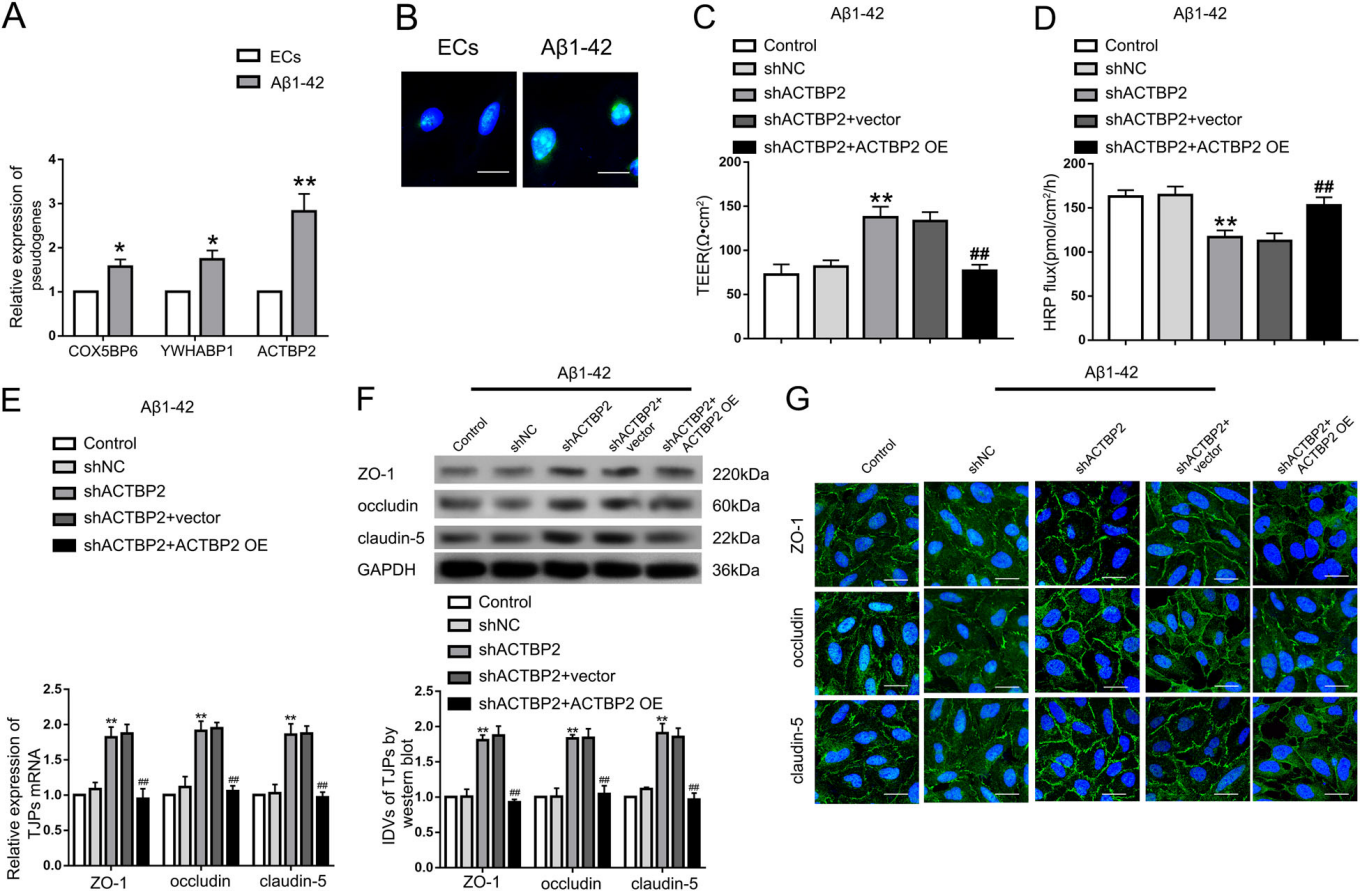
The expression of ACTBP2 in ECs pre-incubated with Aβ_1–42_ and the effect on the BBB permeability in Aβ_1–42_ microenvironment. **A** Relative expression of COX5BP6, YWHABP1, and ACTBP2 in Aβ_1–42_-incubated ECs by qRT-PCR. Data are presented as mean ± SD (*n* = 3), *P < 0.05 versus ECs group, **P < 0.01 versus ECs group. **B** Fluorescence in situ hybridization (FISH) analysis of the location of ACTBP2 (green) mainly in the nucleus of Aβ_1–42_-incubated ECs. Scale bar represents 30 μm. **C**, **D** Effects of ACTBP2 on TEER values (**C**) and HRP flux (**D**) in Aβ_1–42_ microenvironment. **E** Effects of ACTBP2 on ZO-1, occludin, and claudin-5 expression levels in ECs pre-incubated with Aβ_1–42_ determined by qRT-PCR. **F** Effects of ACTBP2 on ZO-1, occludin, and claudin-5 expression levels in ECs pre-incubated with Aβ_1–42_ determined by western blot. Data are presented as mean ± SD (*n* = 3, each). **P < 0.01 versus shNC group. ^##^*P* < 0.01 versus shACTBP2+vector group. **G** Effects of ACTBP2 on ZO-1, occludin, and claudin-5 expression levels and distribution in Aβ_1–42_ microenvironment determined by immunofluorescence staining. ZO-1, occludin, and claudin-5 (green) were labeled with secondary antibodies against anti-ZO-1, anti-occludin, and anti-claudin-5 antibodies, respectively, and nuclei (blue) were labeled with DAPI. Scale bar represents 30 μm.

As shown in Figure S1A, the inhibition rate was increased with Aβ_1–42_ concentration and the time of Aβ_1–42_-incubation. After incubation with 5 μM Aβ_1–42_ for 48 h, the growth of ECs was significantly inhibited, and the inhibition rate was <50%. Thus ECs were incubated with 5 μM Aβ_1–42_ for 48 h in the subsequent experiments. After in vitro BBB model incubated with Aβ_1–42_, transendothelial electric resistance (TEER) values decreased (Figure S1B) and horseradish peroxidase (HRP) flux increased (Figure S1C). It indicated that Aβ_1–42_ might increase the permeability of BBB. To clarify the underlying mechanism of Aβ_1–42_ in the process, the expression levels of tight junction related proteins (TJPs) ZO-1, occludin, and claudin-5 were detected. The expression of the TJPs has decreased in Aβ_1–42_ microenvironment (Figure S1D). Moreover, the images of immunofluorescence assays showed that the TJPs were downregulated in Aβ_1–42_ microenvironment, which exhibited relative discontinuous distribution on the boundaries of Aβ_1–42_-incubated ECs. Pseudogenes microarray was performed to detect housekeeping gene-related pseudogenes, the expression of which changed significantly between normal and Aβ_1–42_-incubated ECs. The housekeeping genes referred to the genes expressed stably in different cells of humans, which were reported by E. Eisenberg et al. [27] based on their RNA-seq results. The expression of ACTBP2 was upregulated most significantly in Aβ_1–42_-incubated ECs by qRT-PCR (Figs. S3A and 1A). The results of fluorescence in situ hybridization (FISH) assays showed that ACTBP2 was distributed in both cytoplasm and nucleus, but the ratio of ACTBP2 in the nucleus was much higher (Fig. 1B). As shown in Fig. 1C and D, TEER values were increased and HRP flux was decreased in shACTBP2 group compared with shNC group. Re-expression of ACTBP2 could rescue the increase in TEER values and the decrease in HRP flux caused by ACTBP2 knockdown. As shown in Fig. 1E and F, mRNA and protein levels of ZO-1, occludin, and claudin-5 in Aβ_1–42_-incubated ECs were promoted after ACTBP2 knockdown, and re-expression of ACTBP2 in the knockdown group attenuated the expression of TJPs compared with shACTBP2+vector group. Consistent with the western blot results, the images of immunofluorescence assays showed that the TJPs were upregulated in the shACTBP2 group, which exhibited relative continuous distribution on the boundaries of Aβ_1–42_-incubated ECs. Re-expressed ACTBP2 in the shACTBP2 ECs reversed these phenotypes (Fig. 1G). These data showed that ACTBP2 was upregulated in ECs pre-incubated with Aβ_1–42_ and might increase the BBB permeability by regulating ZO-1, occludin, and claudin-5 expression in Aβ_1–42_ microenvironment.

### KHDRBS2 was upregulated in Aβ_1–42_-incubated ECs and might increase BBB permeability in Aβ_1–42_ microenvironment

**Fig. 2:**
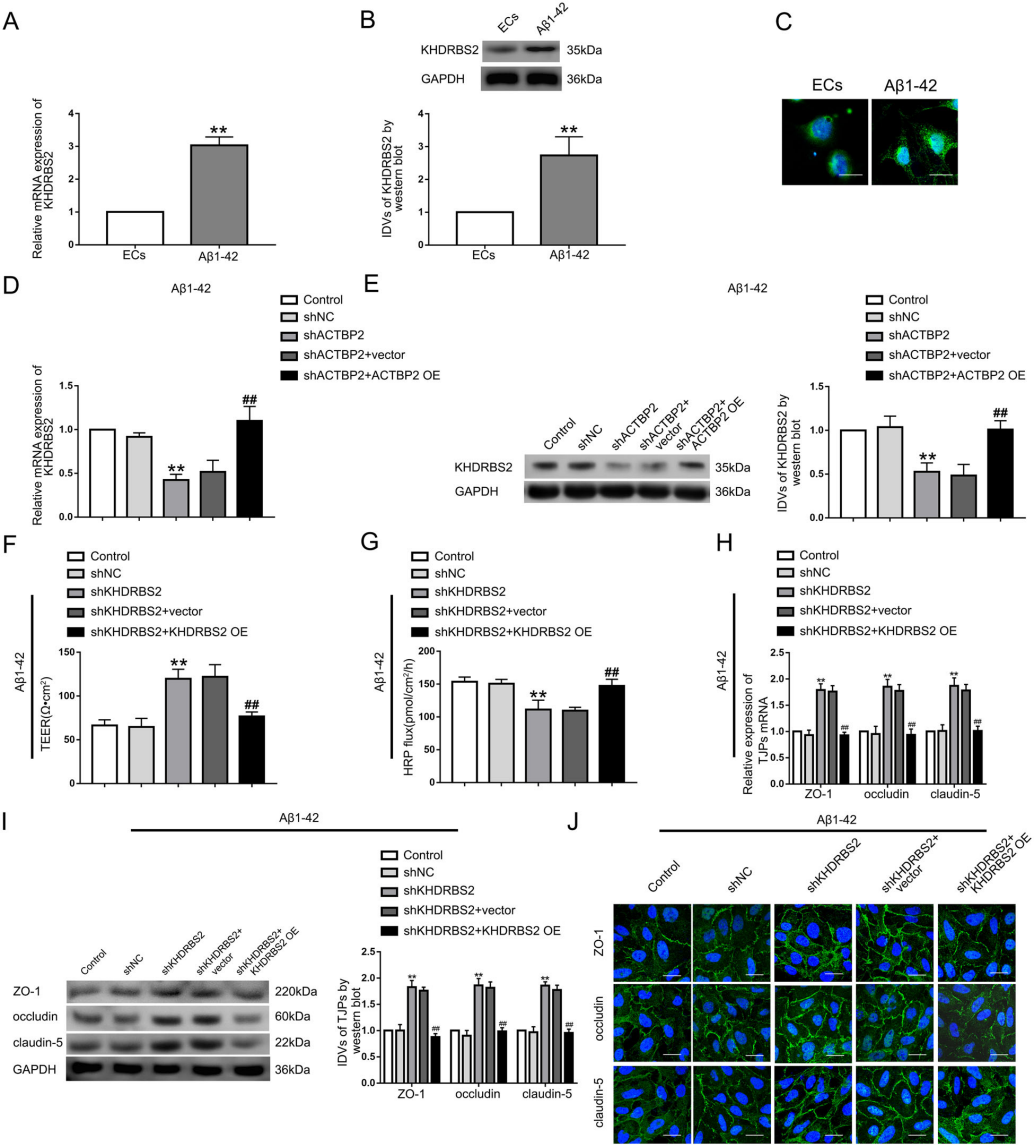
KHDRBS2 endogenous expression and its effects on BBB permeability in Aβ_1–42_ microenvironment. **A** Relative expression of KHDRBS2 mRNA in Aβ_1–42_-incubated ECs by qRT-PCR. **B** Relative expression level of KHDRBS2 protein in Aβ_1–42_-incubated ECs by western blot. Data represent mean ± SD (*n* = 3, each). **P < 0.01 versus ECs group. **C** Immunofluorescence staining analysis of the location of KHDRBS2 (green) in both the nucleus and the cytoplasm of Aβ_1–42_-incubated ECs. Scale bar represents 30 μm. **D** Effects of ACTBP2 on KHDRBS2 mRNA level by qRT-PCR. **E** Effects of ACTBP2 on KHDRBS2 protein level by western blot. Data are presented as mean ± SD (n = 3, each). **P < 0.01 versus shNC group. ^##^*P* < 0.01 versus shACTB*P*2 + vector group. **F**, **G** Effects of KHDRBS2 on TEER values **F** and HRP flux **G** in Aβ_1–42_ microenvironment. **H** Effects of KHDRBS2 on ZO-1, occludin, and claudin-5 expression levels in Aβ_1–42_-incubated ECs determined by qRT-PCR. **I** Effects of KHDRBS2 on ZO-1, occludin, and claudin-5 expression levels in ECs pre-incubated with Aβ_1–42_ determined by western blot. Data are presented as mean ± SD (*n* = 3, each). **P < 0.01 versus shNC group. ^##^*P* < 0.01 versus shKHDRBS2+vector group. **J** Effects of KHDRBS2 on ZO-1, occludin, and claudin-5 expression levels and distribution in Aβ_1–42_ microenvironment by immunofluorescence staining.

We found KHDRBS2 decreased most significantly in shACTBP2 ECs pre-incubated with Aβ_1–42_ (Figure S3B, C) by RNA microarray analysis. The mRNA and protein expression levels of KHDRBS2 were increased significantly in ECs pre-incubated with Aβ_1–42_ compared with normal ECs (Fig. 2A, B). The images of immunofluorescence staining suggested that KHDRBS2 localized in both the nucleus and the cytoplasm (Fig. 2C). KHDRBS2 mRNA and protein levels were decreased in shACTBP2 group, and re-expressed ACTBP2 reversed the changes of KHDRBS2 mRNA and protein expression (Fig. 2D, E). Compared with shNC group, TEER values increased and HRP flux decreased in shKHDRBS2 group, and re-expressing KHDRBS2 reversed the above changes (Fig. 2F, G). The mechanistic studies showed that mRNA and protein levels of ZO-1, occludin, and claudin-5 in Aβ_1–42_-incubated ECs were promoted after KHDRBS2 knockdown, and re-expressing KHDRBS2 decreased the TJPs expression levels compared with shKHDRBS2+vector group (Fig. 2H, I). Similarly, images of immunofluorescence assays showed that KHDRBS2 knockdown induced an increase in the TJPs’ expression. Re-expressing KHDRBS2 reversed the above phenotypes (Fig. 2J). We proposed that KHDRBS2 was highly expressed in ECs pre-incubated with Aβ_1–42_ and might increase BBB permeability in Aβ_1–42_ microenvironment.

### ACTBP2 interacted with KMT2D and WDR5 histone modification complex in the KHDRBS2 promoter in Aβ_1–42_-incubated ECs

**Fig. 3:**
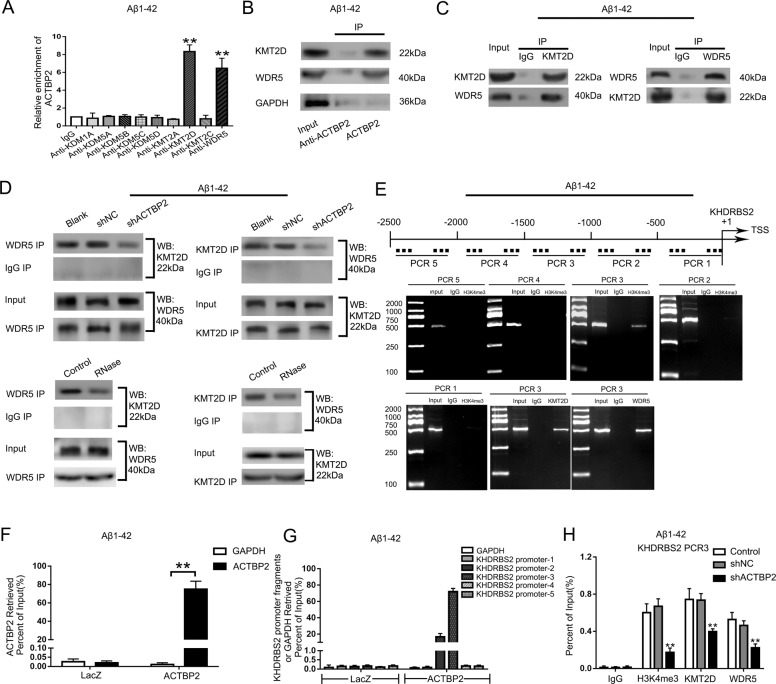
ACTBP2 interacts with KMT2D and WDR5 methyltransferase modification complex in the KHDRBS2 promoter region in Aβ_1–42_-incubated ECs. **A** RNA immunoprecipitation experiments were performed using specific antibodies. Relative enrichment was measured by qRT-PCR. Data are presented as mean ± SD (*n* = 3). **P < 0.01 versus anti-IgG group. **B** Western blot of the proteins from antisense ACTBP2 and ACTBP2 pull-down assays. **C** Co-immunoprecipitation detected the interaction of KMT2D and WDR5 in Aβ_1–42_-incubated ECs. The specific immunoprecipitation of WDR5 and KMT2D was confirmed by western blot. **D** Co-immunoprecipitation assays were performed to detect the interaction between KMT2D and WDR5 after ACTBP2 knockdown (top) or RNase treatment (bottom). **E** KHDRBS2 DNA fragments were detected in the chromatin sample immunoprecipitated from Aβ_1–42_-incubated ECs using the antibody against H3K4me3, KMT2D, or WDR5. **F**, **G** ChIRP analysis of ACTBP2 binding to the KHDRBS2 promoters is shown. **F** The fold enrichment of ACTBP2 in ACTBP2 ChIRP analysis with DNA antisense oligos (asDNA) specific for ACTBP2; **G** the fold enrichment of KHDRBS2 promoter fragments in ACTBP2 ChIRP analysis. LacZ RNA used as the mock control. (*n* = 3) **H** H3K4me3, KMT2D, and WDR5 levels in KHDRBS2 promoter region from 1500 to 1000 bp upstream of the transcription start site (TSS) were decreased after ACTBP2 knockdown in Aβ_1–42_-incubated ECs. Data are presented as mean ± SD (*n* = 3). **P < 0.01 versus shNC group.

Emerging evidence suggests that pseudogene is becoming an important participant in the modification of histone, which can recruit methyltransferases to act on the promoter region of the target gene [28]. Interestingly, ACTBP2 localized predominately in the nuclear (Fig. 1B), and the H3K4me3 accumulation in KHDRBS2 promoter region was predicted with ENCODE (Encyclopedia of DNA Elements) project (Figure S4B). We found that ACTBP2 could bind to the (de)methylases of H3K4me3 including KDM1A, KDM5A, KDM5B, KDM5C, KDM5D, KMT2A, KMT2D, KMT2C, and WDR5 using the bioinformatics database RNA-Protein Interaction Prediction (RPISeq) (as the SVM or RF score > 0.5, Figure S4A). RNA Immunoprecipitation (RIP) results showed enrichment of ACTBP2 in the KMT2D and WDR5 immunoprecipitated samples compared to the IgG immunoprecipitated sample. The results indicated that ACTBP2 interacted with KMT2D and WDR5 respectively. KDM1A, KDM5A, KDM5B, KDM5C, KDM5D, KMT2A, and KMT2C could not bind to ACTBP2 (Fig. 3A). Aβ_1–42_-incubated ECs were transfected with biotinylated ACTBP2 and ACTBP2-antisense, and western blot results following RNA pull-down assays showed that ACTBP2 could specifically interact with KMT2D and WDR5 respectively, which is consistent with the results of RIP (Fig. 3B). The expression of KMT2D and WDR5 did not change significantly in Aβ_1–42_-incubated ECs (Figure S5A, B). Co-immunoprecipitation detected the interaction between KMT2D and WDR5 (Fig. 3C). ACTBP2 knockdown had no significant effect on the expression of KMT2D and WDR5 (Figure S5C, D). ACTBP2 knockdown or RNase treatment of the immunoprecipitation products abrogated the interaction between KMT2D and WDR5 (Fig. 3D). The KHDRBS2 promoter region 2500 bp upstream of the transcription start site (TSS) was divided into five fragments, each containing 500 bp. The DNA fragments were amplified by PCR using their specific primers (Table S5). The results showed that there was H3K4me3 accumulation in the region 1000–1500 bp upstream of the TSS in the KHDRBS2 promoter (PCR3), which were aligned with the prediction results with ENCODE project (Figure S4B). There were immunoprecipitation products of KMT2D and WDR5 in the same promoter region (PCR3), suggesting that KMT2D and WDR5 could bind to KHDRBS2 promoter region (PCR3), respectively (Fig. 3E). Co-immunoprecipitation results showed that both KMT2D and WDR5 could bind to H3K4me3 (Figure S5E, F). The results of chromatin immunoprecipitation (ChIP)-qPCR indicated that KMT2D knockdown and WDR5 knockdown decreased the expression of H3K4me3 in the KHDRBS2 promoter region in Aβ_1–42_ microenvironment (Figure S5G, H). The results are consistent with the previous reports that KMT2D and WDR5 are H3K4 specific methyltransferases and promote the formation of H3K4me3 [15, 29–31]. Next, chromatin isolation by RNA Purification (ChIRP) assays was performed. The biotinylated ACTBP2 probe captured KHDRBS2 promoter-3 in immunoprecipitates determined by qRT-PCR. This suggested that ACTBP2 could bind to the KHDRBS2 promoter region 1000–1500 bp upstream of the TSS (PCR3) (Fig. 3F, G). After ACTBP2 knockdown, H3K4me3 in the KHDRBS2 promoter region decreased significantly, and so did KMT2D and WDR5 (Fig. 3H). The above results indicated that ACTBP2 recruited KMT2D and WDR5 to act on the KHDRBS2 promoter region, catalyzing the formation of H3K4me3. It was confirmed that KMT2D catalyzes tri-methylation of H3K4, and WDR5 is an important cofactor involved in the methylation process [32]. To further confirm that ACTBP2 increases the expression of KHDRBS2 through interacting with methyltransferase complex KMT2D/WDR5, we detect the expression of KHDRBS2 after Aβ_1–42_-incubated ECs were co-transfected with shKMT2D and shACTBP2. The mRNA and protein levels of KHDRBS2 decreased after KMT2D knockdown compared with shNC group (Figure S6A, B), and co-knockdown of ACTBP2 and KMT2D significantly reduced the mRNA and protein levels of KHDRBS2 (Figure S6C, D). KHDRBS2 knockdown enhanced the increase of TEER values and the decrease of HRP flux, which were caused by ACTBP2 knockdown, and KHDRBS2 overexpression rescued the above effects (Figure S6E, F). Similar to the functional studies, TJPs were induced significantly after co-knockdown of ACTBP2 and KHDRBS2, and KHDRBS2 overexpression reversed the shACTBP2-induced increase in the expression of above TJPs (Figure S6G). Thus, ACTBP2 might increase the permeability of BBB in Aβ_1–42_ microenvironment by upregulating the expression of KHDRBS2.

### HEY2 was upregulated in ECs pre-incubated with Aβ_1–42_ and knockdown of HEY2 might attenuate the permeability of BBB in Aβ_1–42_ microenvironment

**Fig. 4:**
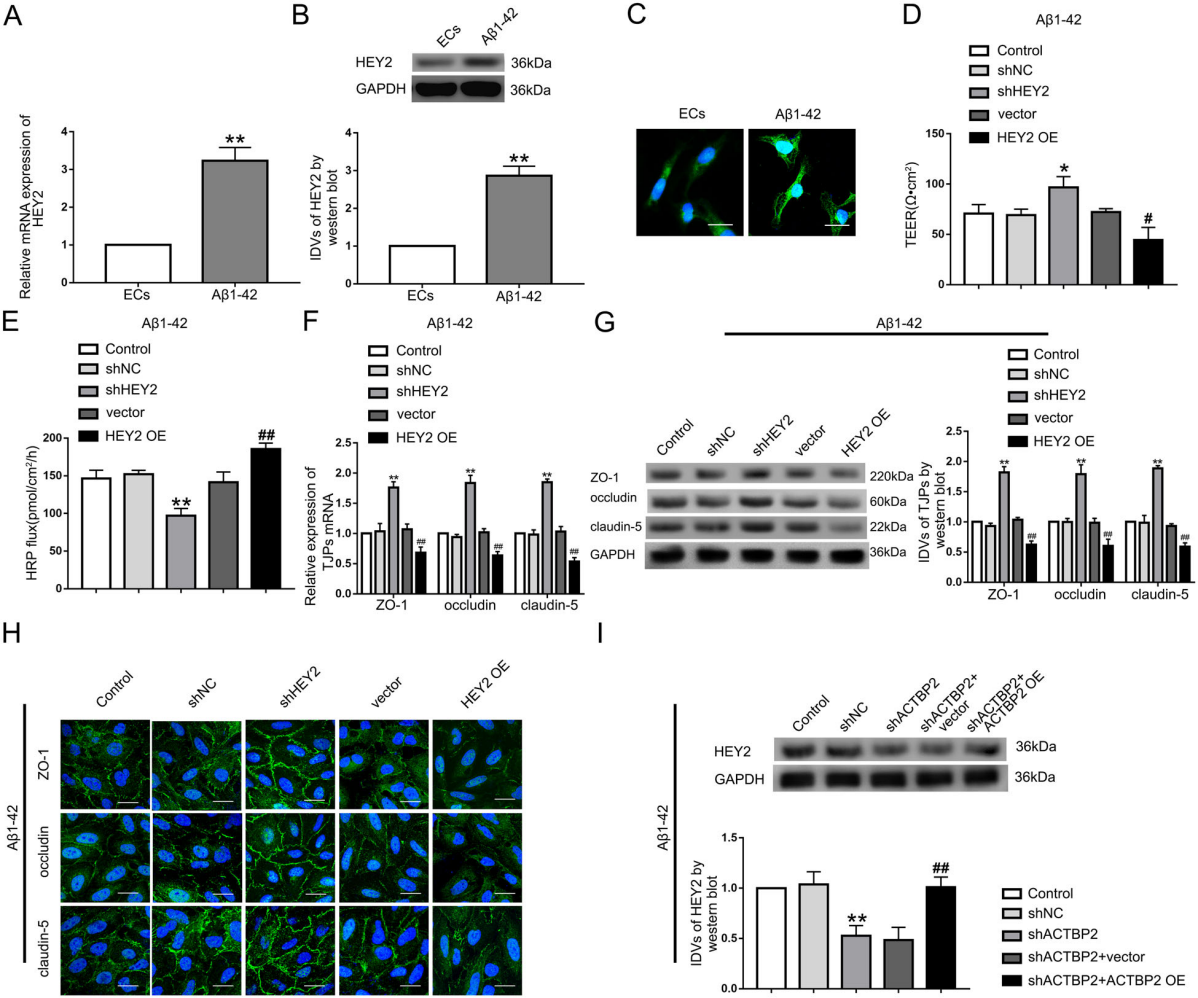
HEY2 was upregulated in Aβ_1–42_-incubated ECs and increased BBB permeability in Aβ_1–42_ microenvironment. **A** Relative mRNA expression level of HEY2 in ECs pre-incubated with Aβ_1–42_ determined by qRT-PCR. **B** Relative protein expression level of HEY2 in ECs pre-incubated with Aβ_1–42_ determined by western blot. Data represent mean ± SD (*n* = 3, each). **P < 0.01 versus ECs group. **C** Immunofluorescence staining analysis of the location of HEY2 (green) in both the nucleus and the cytoplasm of Aβ_1–42_-incubated ECs. Scale bar represents 30 μm. **D**, **E** Effects of HEY2 on TEER values (**D**) and HRP flux (**E**) in Aβ_1–42_ microenvironment. **F** Effects of HEY2 on ZO-1, occludin, and claudin-5 expression levels in Aβ_1–42_-incubated ECs determined by qRT-PCR. **G** Effects of HEY2 on ZO-1, occludin, and claudin-5 expression levels in ECs pre-incubated with Aβ_1–42_ determined by western blot. Data represent mean ± SD (*n* = 3, each). *P < 0.05 versus shNC group. **P < 0.01 versus shNC group. ^#^*P* < 0.05 versus vector group. ^##^*P* < 0.01 versus vector group. **H** Immunofluorescence assays were used to determine ZO-1, occludin, and claudin-5 (green) expression levels and distribution, respectively, in Aβ_1–42_ microenvironment. Scale bar represents 30 μm. **I** Effects of ACTBP2 on HEY2 expression by western blot. Data are presented as mean ± SD (*n* = 3). **P < 0.01 versus shNC group. ^##^*P* < 0.01 versus shACTBP2+vector group.

Using Starbase database and RNAs microarray analysis, we found that many mRNAs interacted with KHDRBS2, and we found HEY2 was downregulated most significantly in shKHDRBS2 ECs in Aβ_1–42_ microenvironment (Figure S7A, S3D, E). The mRNA and protein levels of HEY2 were induced in ECs pre-incubated with Aβ_1–42_ (Fig. 4A, B). HEY2 was located in both nucleus and cytoplasm detected by immunofluorescence staining (Fig. 4C). The shHEY2 group exhibited the increase in TEER values and the decrease in HRP flux, and the opposite effects were shown in HEY2 OE group (Fig. 4D, E). The mRNA and protein levels of ZO-1, occludin, and claudin-5 were increased in shHEY2 group, and inhibited by HEY2 overexpression (Fig. 4F, G). Similarly, the above proteins exhibited higher expression with relative continuous distribution in shHEY2 group compared with shNC group and lower expression with relative discontinuous distribution in HEY2 OE group compared with vector group (Fig. 4H). ACTBP2 knockdown inhibited the HEY2 expression, and re-expression of ACTBP2 reversed the reduction (Fig. 4I). The results indicated that HEY2 might increase the permeability of BBB in Aβ_1–42_ microenvironment.

### KHDRBS2 might decrease the permeability of BBB through stabilizing HEY2 mRNA in Aβ_1–42_ microenvironment

**Fig. 5:**
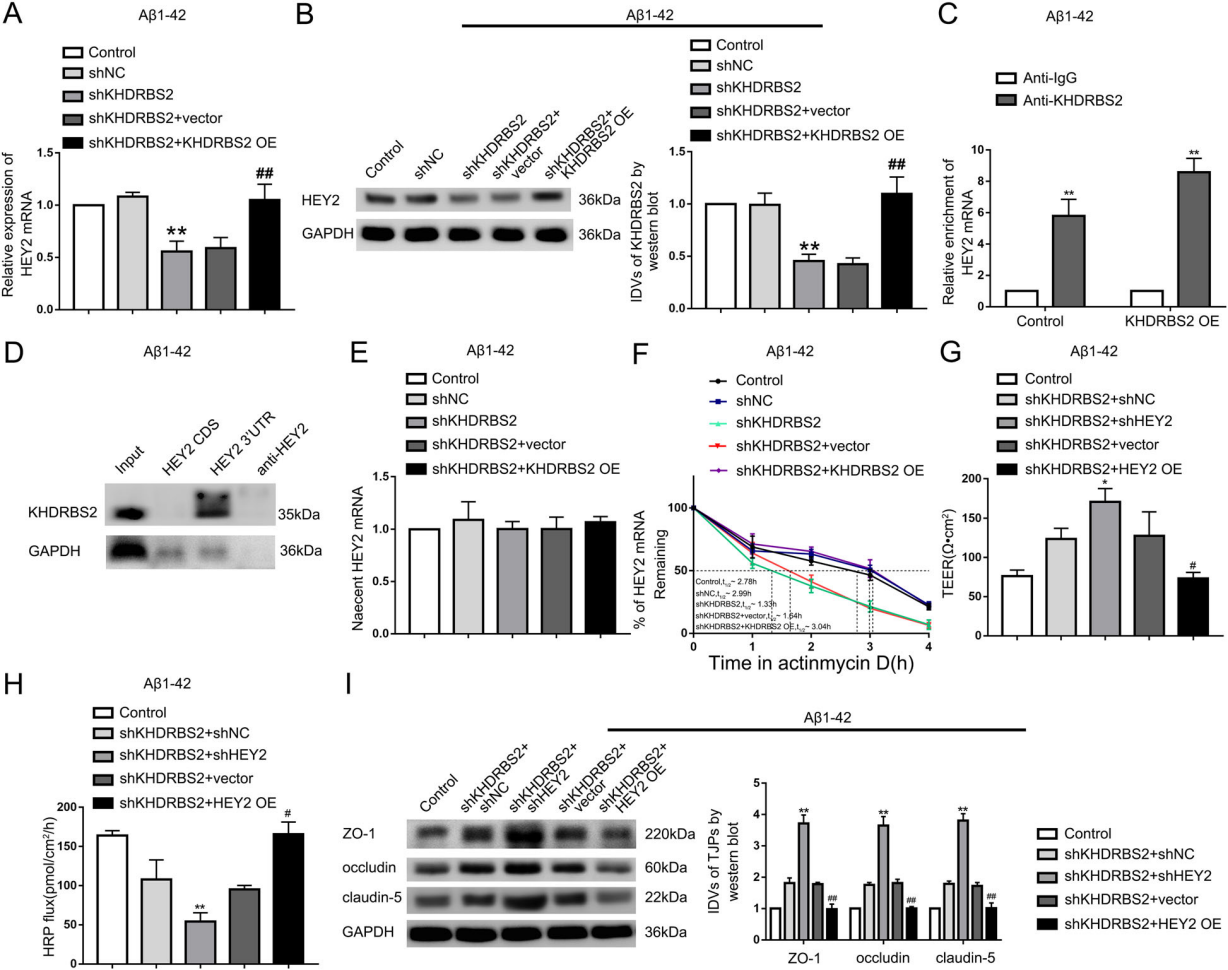
KHDRBS2-regulated BBB permeability by increasing the half-life of HEY2 mRNA in Aβ_1–42_ microenvironment. **A** Effects of KHDRBS2 on HEY2 mRNA expression in ECs pre-incubated with Aβ_1–42_ by qRT-PCR. **B** Effects of KHDRBS2 on HEY2 protein expression in ECs pre-incubated with Aβ_1–42_ by western blot. Data are presented as mean ± SD (*n* = 3, each). **P < 0.01 versus shNC group. ^##^*P* < 0.01 versus shKHDRBS2+vector group. **C** RNA immunoprecipitation confirmed the bonding between KHDRBS2 and HEY2 mRNA in Aβ_1–42_ microenvironment. Relative enrichment was measured by qRT-PCR. Data are presented as mean ± SD (*n* = 3, each). **P < 0.01 versus Anti-IgG group. **D** Western blot of the proteins from HEY2 CDS, HEY2 3′UTR, and antisense HEY2 pull-down assays in Aβ_1–42_ microenvironment. **E** Nascent HEY2 levels in Aβ_1–42_-incubated ECs by qRT-PCR. Data are presented as mean ± SD (*n* = 3, each). **F** The graph shows HEY2 levels in ECs pre-incubated with Aβ_1–42_ at different times treated with ActD in the Control, shNC, shKHDRBS2, shKHDRBS2+vector, and shKHDRBS2 + KHDRBS2 OE groups by qRT-PCR. **G**, **H** Effects of KHDRBS2 knockdown and HEY2 on TEER values **G** and HRP flux **H** in Aβ_1–42_ microenvironment. **I** Effects of KHDRBS2 knockdown and HEY2 on ZO-1, occludin, and claudin-5 expression levels in Aβ_1–42_-incubated ECs by western blot. Data are presented as mean ± SD (*n* = 3, each). *P < 0.05 versus shKHDRBS2+shNC group, **P < 0.01 versus shKHDRBS2+shNC group, ^#^*P* < 0.05 versus shKHDRBS2+ vector group, ^##^*P* < 0.01 versus shKHDRBS2+vector group.

The mRNA and protein levels of HEY2 were inhibited by KHDRBS2 knockdown, and re-expressing KHDRBS2 reversed the phenotype (Fig. 5A and B). RIP results indicated that KHDRBS2 could bind to HEY2 mRNA in Aβ_1–42_ microenvironment, and overexpression of KHDRBS2 increased the immunoprecipitation of anti-KHDRBS2 group (Fig. 5C). The results of RNA pull-down showed that KHDRBS2 could bind to the 3′UTR instead of the CDS of HEY2 (Fig. 5D). PCR results after nascent RNA capture assays showed that there was no significant effect of KHDRBS2 on HEY2 mRNA synthesis (Fig. 5E). KHDRBS2 inhibition decreased the half-life of HEY2, and re-expressing KHDRBS2 could reverse the decrease (Fig. 5F). The data suggested that KHDRBS2-regulated HEY2 at a post-transcriptional level. Next, stable shKHDRBS2 ECs were transfected with shHEY2 or HEY2 OE plasmid to investigate the effects of HEY2 on KHDRBS2-regulated the permeability of BBB in Aβ_1–42_ microenvironment. HEY2 inhibition enhanced the increase in TEER values and the decrease in HRP flux which were caused by KHDRBS2 knockdown, and overexpressing HEY2 could reverse above functional changes of BBB (Fig. 5G, H). Similarly, mechanistic studies showed that the increase of ZO-1, occludin, and claudin-5 expression levels caused by KHDRBS2 knockdown were enhanced by HEY2 knockdown, and reversed by HEY2 overexpression (Fig. 5I). These results indicated that KHDRBS2 might increase the permeability of BBB in Aβ_1–42_ microenvironment by increasing the stability of HEY2 mRNA.

### HEY2 transcriptionally inhibited the expression of ZO-1, occludin, and claudin-5 in Aβ_1–42_ microenvironment

**Fig. 6:**
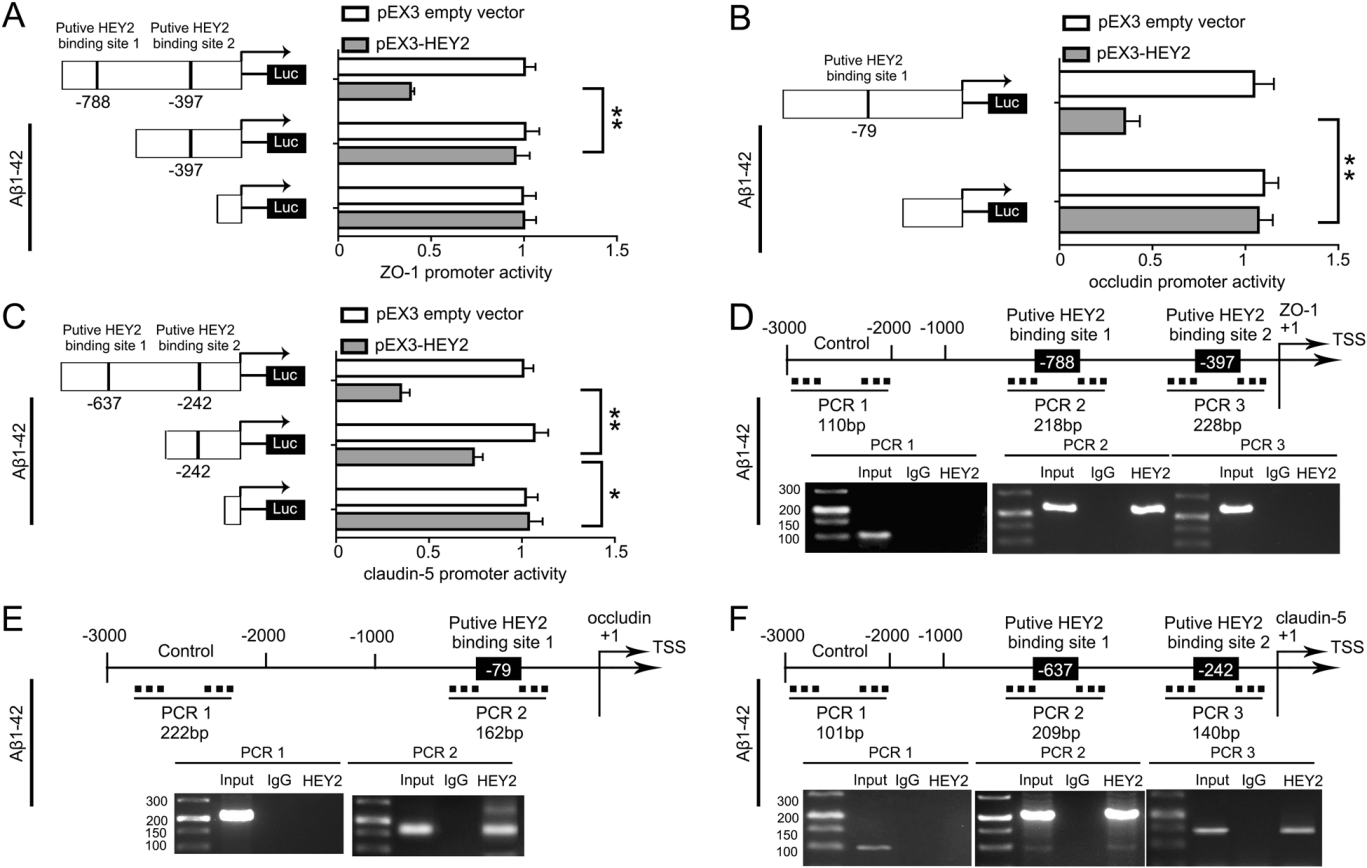
HEY2 decreased the Promoter Activity of ZO-1, occludin, and claudin-5 in ECs pre-incubated with Aβ_1–42_. **A**–**C** Schematic depiction of the different reporter plasmids and relative luciferase activity in Aβ_1–42_ microenvironment. The relative luciferase activity was conducted after Aβ_1–42_-incubated ECs co-transfected with ZO-1 **A**, occludin **B**, or claudin-5 **C** promoter (−1000 to 0 bp) (or promoter-deleted putative HEY2-binding sites) with pEX3-HEY2 or pEX3 empty vector. Data represent mean ± SD (*n* = 3, each). **P < 0.01, *P < 0.05. **D**–**F** HEY2 interacted with the promoters of ZO-1 **D**, occludin **E**, and claudin-5 **F** in Aβ_1–42_-incubated ECs. The transcription start site (TSS) was designated as +1. Putative HEY2-binding sites are illustrated. Immunoprecipitated DNA was amplified by PCR. Normal rabbit IgG was used as a negative control.

Using JASPAR database, the promoters of ZO-1, occludin, and claudin-5 was predicted to have putative binding sites of HEY2 (Figure S7B–D). Luciferase reporter assays and ChIP assays were performed to verify the possibilities of HEY2 regulating ZO-1, occludin, and claudin-5 at the transcriptional level in Aβ_1–42_ microenvironment. By analyzing the DNA sequence of ZO-1 promoter in the region 1000 bp upstream of TSS, we identified two binding sites of HEY2. Deletion of the putative binding site 1 (−788bp site region) significantly increased the promoter activities of ZO-1 (Fig. 6A). Similarly, deletion of the putative binding site 1 (−79bp site region) significantly increased the promoter activities of occludin (Fig. 6B). The promoter activities of claudin-5 were increased after deletion of putative binding site 1 (−637bp site region) or putative binding site 2 (−242bp site region) (Fig. 6C). ChIP assays were carried out to verify the above results. Primers for the putative binding sites (Table S4) designed, and the binding DNA fragments were amplified by PCR. The presence of immunoprecipitates indicated the same results as the luciferase assays. Immunoprecipitates of HEY2 and the putative binding site 1 (−788bp site region) were observed in PCR2 products (Fig. 6D), indicating that HEY2 bond to the putative binding site 1 of ZO-1. Similarly, HEY2 bond to putative binding site 1 of occludin (Fig. 6E), putative binding sites 1 and 2 of claudin-5 (Fig. 6F). These results suggested that HEY2 increased the permeability of BBB as a transcriptional inhibitor of ZO-1, occludin, and cluadin-5 in Aβ_1–42_ microenvironment.

### Both Khdrbs2- and Hey2 knockdown resulted in upregulation of ZO-1, occludin, and claudin-5 in APP/PS1 transgenic mice cerebral microvascular ECs

**Fig. 7:**
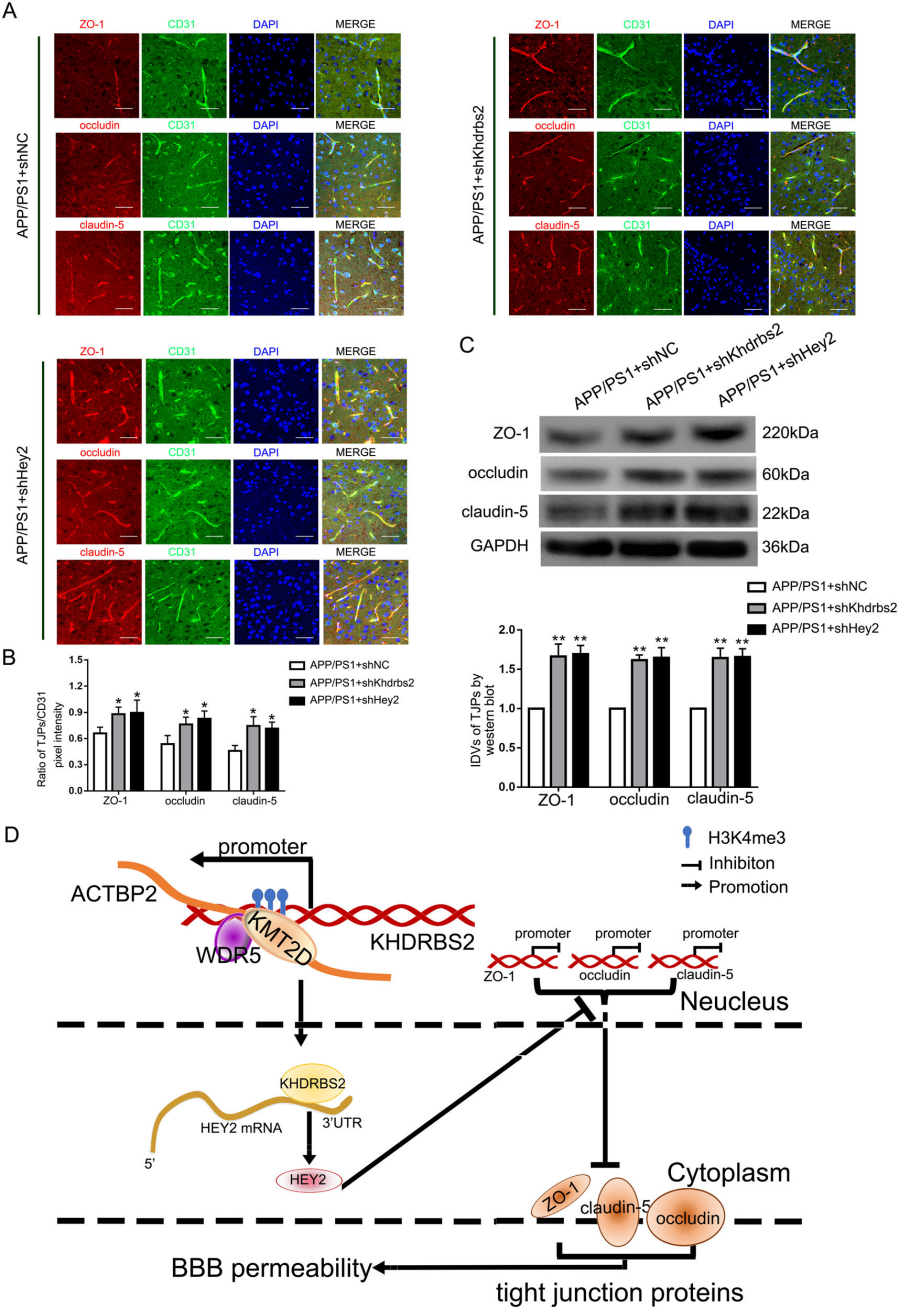
Increase of ZO-1, occludin, and claudin-5 expression caused by Khdrbs2 knockdown or Hey2 knockdown in APP/PS1 mice brain microvessels. **A** Immunohistofluorescence staining of ZO-1, occludin, and claudin-5 expression in vivo. Frozen slices (10 μm thick) of the corpus striatums from APP/PS1 + shNC, APP/PS1 + shKhdrbs2, and APP/PS1 + shHey2 groups were stained with CD31 (green), TJPs (red), and DAPI (blue). Scale bar represents 100 μm. **B** Quantification of pixel intensity. (*n* = 5) **C** The effects of Khdrbs2 knockdown or Hey2 knockdown on APP/PS1 brain microvessels. Data are presented as mean ± SD (*n* = 3). *P < 0.05 versus APP/PS1 + shNC group, **P < 0.01 versus APP/PS1 + shNC group. **D** The schematic illusion of interactions between ACTBP2, KHDRBS2, and HEY2 in Aβ1–42-incubated ECs.

To verify the effect of Khdrbs2 or Hey2 on the expression of ZO-1, occludin, and claudin-5 in vivo, shKhdrbs2 and shHey2 were constructed in the adeno-associated viral vectors (AAVs) respectively. We used AAV2/9 serotype that can be transfected into mice cerebral microvessels. Immunohistofluorescence staining of corpus striatum showed that knockdown of Khdrbs2 or Hey2 increased the fluorescence intensity of ZO-1, occludin, and claudin-5 compared with APP/PS1 + shNC group, suggesting that the expression levels of TJPs were increased (Fig. 7A and B). Similarly, western bolt results of the protein from the mice microvessels revealed higher levels of ZO-1, occludin, and claudin-5 in APP/PS1 + shKhdrbs2 and APP/PS1 + shHey2 groups compared with APP/ PS1 + shNC group (Fig. 7C). These results indicated that both Khdrbs2 knockdown and Hey2 knockdown could increase the expression of ZO-1, occludin, and claudin-5 in AD transgenic mice microvascular ECs.

## Discussion

BBB dysfunction is a pathological change in the early stage of AD. The transition between the dysfunction of ECs and the deposition of Aβ in neurons promotes the development of AD [7]. More and more evidences show that pseudogenes play an important role in the progression of AD and endothelial function [33, 34]. ACTBP2 is produced by transcribing DNA into mRNA, then reverse transcribing it into cDNA and inserting it into a new site in the genome [35–37]. Our study found that ACTBP2 was highly expressed in Aβ_1–42_-incubated ECs compared with normal ECs. ACTBP2 reduced TEER values of BBB and increased HRP flux of BBB, which indicated that the permeability of BBB was increased in Aβ_1–42_ environment. ACTBP2 inhibition increases the expression of ZO-1, occludin, and claudin-5 in ECs pre-incubated with Aβ_1–42_, and decreases the permeability of BBB.

RBPs interact with RNA and play an important role in post-transcriptional regulation and mRNA transport, turnover, storage, and translation. The pathological increase of RBPs may accelerate the pathophysiology of many neurodegenerative diseases including AD [38]. We previously reported that RBP TRA2A increases the permeability of BBB by increasing the stability of LINC00662 in Aβ_1–42_ microenvironment [39]. For the first time, we found that KHDRBS2 was upregulated in Aβ_1–42_-incubated ECs, and KHDRBS2 might increase the permeability of BBB in Aβ_1–42_ microenvironment. It has been reported that KHDRBS2 induces plasma membrane stress by activating TORC2 kinase complex in AD mouse model, increasing Aβ in the brain and reducing cognitive function in AD mice [40, 41]. Our study demonstrates that KHDRBS2 may exacerbate the progression of AD.

We confirmed that ACTBP2 could directly bind to KMT2D and WDR5, respectively, through RIP assay and RNA pull-down assays for the first time. It has been reported that KMT2D, as a specific H3K4 methylase, interacts with WDR5 to catalyze the de/tri-methylation of H3K4 in the promoter region of the target gene, and WDR5 aids this process by directly binding to the amino acids of histone H3 [19, 29–31]. The results of co-immunoprecipitation assays in this study confirmed that KMT2D would interact with WDR5 in ECs under Aβ_1–42_ microenvironment. Accumulation of H3K4me3 was found in the 1000–1500 bp upstream of KHDRBS2 TTS from ChIP assays. This is aligned with the prediction results with ENCODE (Encyclopedia of DNA Elements) database. Interestingly, the interaction between KMT2D and WDR5 decreases significantly in the absence of ACTBP2. It indicates that ACTBP2 is required for the bonding of KMT2D and WDR5 in Aβ_1–42_-incubated ECs. ChIRP experiments confirmed that ACTBP2 bond to the KHDRBS2 promoter. Knocking down of ACTBP2 can reduce the expression of KMT2D, WDR5, and H3K4me3 in KHDRBS2 promoter. The experiment results show that ACTBP2 promotes the interaction between KMT2D and WDR5, recruits KMT2D/WDR5 to the KHDRBS2 promoter, generates H3K4me3 in KHDRBS2 promoter, and promotes the transcription of KHDRBS2 in AD environment. However, the mechanism of ACTBP2 recruiting KMT2D/WDR5 to KHDRBS2 promoter is not clear until now. Previous studies have demonstrated that pseudogenes are involved in histone modification and affect the expression of downstream genes. For example, pseudogene PTENpg1 antisense RNA α recruits histone methyltransferases EZH2 and DNMT3A and anchors them to the PTEN promoter to regulate PTEN transcription by regulating histone modification in HEK293 cells [28]. The results of this study suggest that ACTBP2 may promote KHDRBS2 transcription by regulating histone modification in the KHDRBS2 promoter region.

HEY2 is a member of the bHLH transcription repressor family [23]. This study demonstrated that HEY2 is highly expressed in Aβ_1–42_-incubated ECs, and might increase the permeability of BBB by reducing the expression of ZO-1, occludin, and claudin-5 in Aβ_1–42_ microenvironment. The results of ChIP assays and luciferase experiments indicated that HEY2 could bind to the promoter regions of the TJPs, and inhibit the expression of mRNA and protein of these three genes. Similar to the results of HEY2 promoting AD progression in this study, activation of Notch signaling pathway in rat hippocampal neurons cells can lead to the increase of HEY2 translation and decrease of cognitive function of elderly rats [42]. It is reported that HEY2 binds to Smad3 and Smad4 promoters to inhibit the transcriptional activity of Smad3 and Smad4, and accelerates the malignant progression of hepatocellular carcinoma through TGF-β/Smad signaling pathway [43]. Taken together, HEY2 may promote AD progression by transcriptional inhibition of the expression of ZO-1, occludin, and claudin-5 in ECs in BBB.

RBPs can regulate the subcellular localization, half-life, or translation speed of mRNA by binding to mRNA 3′UTR [44, 45]. In this study, we found that KHDRBS2 directly bonds to the 3′UTR of HEY2 mRNA, and knockdown of KHDRBS2 reduces the stability of HEY2 mRNA. KHDRBS2 increases BBB permeability in Aβ_1–42_ microenvironment by increasing the stability of HEY2. Similarly, in AD mice’s neurons, RBP HUD bonds with APP mRNA and BACE1 mRNA 3′UTRs, increases the stability of APP and BACE1 mRNA, and promotes the production of Aβ, and accelerated the progression of AD [46]. It has been reported that RBPs regulate the function of ECs by regulating mRNA stability. For example, in the human umbilical vein ECs (HUVECs) in rheumatoid arthritis model, bonding of RBP TTP and intercellular cell adhesion molecule-1 (ICAM-1) mRNA 3′UTR decreases the stability of ICAM-1, reduces the expression of ICAM-1 in HUVECs, and participates in the inhibition of inflammatory response of rheumatoid arthritis [47]. In HUVECs treated with high glucose, bonding of HuR and Sirtuin 1 (SIRT1) mRNA increases the stability of SIRT1 mRNA, thus inhibits E-selectin release and the activation of ECs induced by inflammation and hyperglycemia [48]. Combining these study results, KHDRBS2 may increase BBB permeability in Aβ_1–42_ microenvironment by directly regulating the expression of HEY2.

Finally, as ACTBP2 has no homologous gene in mice, we demonstrated that knocking down of Khdrbs2 or knocking down of Hey2 significantly increases the expression of ZO-1, occludin, and claudin-5 in APPswe/PS1dE9 transgenic mice brain ECs. ACTBP2, KHDRBS2, and HEY2 play an important role in the development of AD, and may become potential new targets for the treatment of AD.

In summary, we demonstrated that ACTBP2, KHDRBS2, and HEY2 were upregulated in ECs in Aβ_1–42_ microenvironment for the first time. Knocking down of ACTBP2, KHDRBS2, or HEY2 reduces the BBB permeability in Aβ_1–42_ microenvironment. ACTBP2 recruits KMT2D and WDR5, promotes the bonding of KMT2D and WDR5, and anchors the KMT2D/WDR5 methyltransferase complex to the KHDRBS2 promoter, promotes the creation of H3K4me3 near the transcription starting sites, enhances KHDRBS2’s transcription activity, and increases the expression of KHDRBS2. KHDRBS2 increases HEY2 mRNA stability by binding to its 3′UTR and promotes the expression of HEY2. This research revealed that ACTBP2/KHDRBS2/HEY2 module regulated BBB permeability in Aβ1–42 microenvironment, and presented a new direction and experimental method for the study of AD development.

## Materials and methods

### Cell cultures

The immortalized human brain EC line hCMEC/D3 was acquired from Dr. Couraud (Institute Cochin, Paris, France). Human brain vascular pericytes and normal human astrocytes were purchased from the Sciencell Research Laboratories (Carlsbad, CA, USA). Human embryonic kidney 293 (HEK293T) cells were purchased from Shanghai Institutes for Biological Sciences Cell Resource Center. All the cells were cultured in a humidified atmosphere (37°C, 5% CO_2_) as previously detailed [39]. ECs were limited from 30 to 35 passages. ECs were pre-incubated with Aβ_1–42_ (5 μM) for 48 h as previously detailed [39].

### Animals and experiment design

The APPswe/PSEN1dE9 (APP/PS1) transgenic male mice in C57BL6 mice background were originally generated by and purchased from the Jackson Laboratory (West Grove, PA). The experiment of APP/PS1 mice was carried out in accordance with“Laboratory Animals-Guideline of welfare and ethics, The Ethics Committee for Medical Laboratory Animals of China Medical University”. Efforts were made to reduce the mice suffering and the number of mice used.

All mice were fed in a controlled environment (50% humidity, 22–25°C, 12 h light/dark cycle, lights on at 08:00) with standard mouse diet and water. Twenty-four male mice (6-month old) were divided into three groups randomly: APP/PS1 + shNC group (*n* = 8), APP/PS1 + shKhdrbs2 group (*n* = 8), and APP/PS1 + shHey2 group (*n* = 8). For mouse studies, *n* = 5 mice per group were used for Immunofluorescence staining experiments, and *n* = 3 mice per group were used for western blot assays. The investigators were blinded to the group allocation during the experiment and when assessing the outcomes of immunohistofluorescence staining and western blot.

### Intracerebraoventricular injection

Recombinant AAV2/9 was used to repress the gene expression in APP/PS1 mice cerebral microvascular ECs. Short-hairpin RNA directed against mouse Khdrbs2 (NM_133235.3), Hey2 (NM_013904.1), and control shRNA sequences were ligated into pAKD-CMV-bGlobin-eGFP-H1-shRNA (Obio Technology, Shanghai, China). Sequences of shKhdrbs2 and shHey2 were shown in Table S3. The mice were anesthetized with 1.5% isoflurane through intravenous injection and an operation was performed after the mice were completely static and unresponsive to a toe pinch. The intracerebraoventricular injections of the virus to transfect the mouse cerebral microvascular ECs were performed using a stereotaxic apparatus as described previously [49]. The expression of ZO-1, occludin, and claudin-5 were observed 4 weeks after the virus injection. The transfection efficiency was shown in Figure S8.

### In vitro BBB models establishment

In vitro co-culturing BBB models in Aβ_1–42_ microenvironment were established as previously described [39]. For details, see Supplemental Materials and Methods.

### Growth inhibition assays

See Supplemental Materials and Methods.

### Real-time PCR assays

Cell lines RNA was extracted by Trizol Reagent (Life Technologies Corporation, Carlsbad, CA, USA). See Supplemental Materials and Methods.

### Cell transfections

See Supplemental Materials and Methods.

### TEER assays

TEER assays were performed using a millicell-ERS apparatus (Millipore, Billerica, MA, USA) after in vitro BBB models constructed. TEER values were measured by the apparatus at room temperature after exchanging the medium. Electric resistances were obtained by subtracting the background values from the measured values. TEER value (Ω cm^2^) is electric resistance multiplied by the effective surface area of the chamber of the transwell system.

### HRP flux assays

1 ml of serum-free EBM-2 medium containing 10 μg/mL HRP (0.5 mmol/L, Sigma-Aldrich) was added into the upper chamber of the transwell system after in vitro BBB models constructed. In all, 5 μL of culture medium in the lower chamber was collected after the medium was placed for 1 h. Using the tetramethylbenzidine colorimetry approach, the HRP content of the samples was measured. The final HRP value was expressed as pmol/cm^2^/h.

### Western blot assays

Microvessels were isolated from the transfected APP/PS1 mice following previously published methods [50]. Western blot assays were established as previously described [39]. See Supplemental Materials and Methods for details.

### Cell immunofluorescence staining

Immunofluorescence assays were established as previously described [39]. See Supplemental Materials and Methods for details.

### Immunohistofluorescence staining

See Supplemental Materials and Methods for details.

### Fluorescence in situ hybridization

To determine ACTBP2 location in Aβ_1–42_-incubated ECs, FISH assays were performed with ACTBP2 probe (5′-TTGCTGAGCTACTTTGTATT-3′) as previously described [39]. See Supplemental Materials and Methods for details.

### Chromatin immunoprecipitation assays

The Simple-ChIP Enzymatic Chromatin IP Kit (Cell Signaling Technology, Danvers, USA) was used to perform ChIP assays as previously described [39]. The antibodies used in assays were as follows: anti-HEY2 antibody (Cat# 10597, Proteintech), anti-H3K4me3 antibody (Cat# 61379, Proteintech), anti-KMT2D (Cat# 27266-1-AP, Thermo Fisher Scientific) and anti-WDR5 (Cat# MA5–32760, Thermo Fisher Scientific) and normal rabbit IgG. See Supplemental Materials and Methods for details.

### Human pseudogenes and RNA microarrays

Pseudogenes and RNA analysis, sample preparation, and microarray hybridization were completed by Kangchen Bio-tech (Shanghai, China).

### Reporter vector construction and luciferase reporter assays

See Supplemental Materials and Methods for details.

### RIP assays

RIP assays were performed using EZ-Magna RBP immunoprecipitation kit (Millipore, USA) according to the manufacture’s protocol. See Supplemental Materials and Methods for details.

### RNA pull-down assays

See Supplemental Materials and Methods for details.

### Co-immunoprecipitation

Pierce co-immunoprecipitation (Co-IP) Kit (Cat# 26149, Thermo Fisher Scientific) was used to perform Co-IP as previously described [51]. Both input proteins and IP proteins were detected using a standard western blot technique.

### Chromatin Isolation by RNA Purification assays

ChIRP assays was performed as previously described [52]. See Supplemental Materials and Methods for details.

### Nascent RNA capture

Click-iT® Nascent RNA Capture Kit (Cat# C10365, Thermo Fisher Scientific, USA) was used to detect Nascent RNAs according to the manufacture’s protocol. Nascent RNAs were marked with 0.2 mM 5-ethymyl uridine (EU) and the EU-nascent RNA was captured on magnetic beads. Nascent RNAs were detected using qRT-PCR.

### mRNA stability assays

In all, 5 μg/ml actinomycin D (ActD, NobleRyder, China) was added into ECs culture medium to inhibit the nascent RNA synthesis. Total RNA was extracted at 0, 1, 2, 3, 4 h after ActD was added. HEY2 mRNA was detected by qRT-PCR, and the mRNA half-life was the time taken for the concentration to fall to half of its original value.

### Statistical analysis

Data meet the normal distribution and the variance is similar. Data are described as mean ± standard deviation. All differences were analyzed by SPSS 18.0 statistical software with the Student’s *t* test (two-tailed) or one-way analysis of variance. *P* < 0.05 was considered significant.

## Supplementary information

Supplemental Material and Methods

Figure S1

Figure S2

Figure S3

Figure S4

Figure S5

Figure S6

Figure S7

Figure S8

Supplemental Tables

Spplemental Figure legends

## Data Availability

The data supporting the conclusion of this research have been included in this published article and its additional files.
