# Supplementary material for: Pseudogene ACTBP2 increases blood–brain barrier permeability by promoting KHDRBS2 transcription through recruitment of KMT2D/WDR5 in Aβ1–42 microenvironment
Source: Cell Death Discov. 2021 Jun 14;7:142. doi: 10.1038/s41420-021-00531-y (PMC8203645; doi:10.1038/s41420-021-00531-y)
Supplement: Supplementary file 1 — Supplemental Material and Methods [file 41420_2021_531_MOESM1_ESM.docx]

**Supplemental Materials and Methods**

***In vitro* BBB models establishment**

First, after pericytes were cultured (2×10^5^ cells/cm^2^) on the lower chambers of Transwell inserts (0.4 μm pore size; Corning, NY, USA) overnight, hCMEC/D3 cells were subsequently placed on the upper chambers of Transwell inserts. NHA (2×10^5^ cells/cm2) were seeded onto the 6-well culture plate and cultured for 2 days before adding ECs inserts.

**Real-time PCR assays**

The RNA concentration and quality were detected by Nanodrop Spectrophotometer (ND-100, Thermo Scientific). The expression levels of ACTBP2 (NG_003019.6), KHDRBS2 (NM_001350622.2), and HEY2 (NM_012259.3) were detected by One-Step SYBR PrimeScript RT-PCR Kit (Perfect Real Time; Takara Bio, Inc., Japan). Relative expression values were calculated using the relative quantification (2^−△△Ct^) method. Primers are shown in Table S1.

**Cell transfections**

Short-hairpin RNA (shRNA) directed against ACTBP2 (NG_003019.6) was ligated into LV10 (U6/GFP&Neo) vector (GenePharma, Shanghai, China) to construct the shACTBP2 plasmid. The plasmid with ACTBP2 full-length was ligated into pGCMV/MCS/IRES/EGFP/Neo (GenePharma, Shanghai, China) to construct the ACTBP2 (O/E) plasmid. ShRNA directed against human KHDRBS2 (NM_001350622.2), HEY2 (NM_012259.3), KMT2D (NM_003482.4), and WDR5 (NM_001384409) genes were ligated into the pGPU6/GFP/Neo vector (GenePharma) to construct shKHDRBS2, shHEY2, shKMT2D and shWDR5 plasmids, respectively. The human KHDRBS2 and HEY2 full-length sequence were ligated into pIRES2 vector (GenScript, Piscataway, USA) to construct the KHDRBS2(O/E) and HEY2(O/E) vectors. The respective no-targeting sequences were used as shNC or vectors. The ECs were stable transfected *via* LTX and Plus reagent (Life Technologies, USA) in AD microenvironment. The stable transfected cells were selected using G418 (0.4 mg/ml) (Sigma-Aldrich, USA). Sequences of shACTBP2, shKHDRBS2, shHEY2, shKMT2D, and shHEY2 were shown in Table S2. For co-transfection of shACTBP2 and ACTBP2 (O/E), ACTBP2(O/E) plasmids were transiently transfected into Aβ_1-42_-incubated ECs, which were stably transfected with shACTBP2. After 48 hours, the transiently transfected cells were obtained. Other co-transfected ECs were established in the same way in AD microenvironment. The transfection efficiency were measured by qRT-PCR and western blot, and were shown in Figure S2.

**Growth inhibition assays**

Cells were seeded at 2000 per well in 96-well plates with 200 μL serum free EBM-2 medium, incubated for 24 h and treated with various treatments for different time. Blank wells had only

serum free medium without cells or treatments. The cell inhibition rate was assayed using the Cell Counting Kit-8 (CCK-8, Beyotime Institute of Biotechnology, Jiangsu, China) according to the instruction provided by the manufacturer. 10 μL CCK-8 was added into each well and cells were incubated for another two hours in a humidified incubator. Optical density value was measured at

450 nm. The cell inhibition rate was calculated from the following formula:

Inhibition rate (%) = (OD_450_ of control cells-OD_450_ of treated cells) / (OD_450_ of treated cells-OD_450_ of blank cells) x100%.

Five replicated wells were set up in each group and independently repeated experiments were performed three times.

**Western blot assays**

ECs or microvessels extracted from mice brain were lysed in PIPA buffer (Beyotime, China). Total proteins were extracted with RIPA buffer (Beyotime, China) supplemented with protease inhibitors (10 mg/mL aprotinin, 10 mg/mL phenyl-methylsulfonyl fluoride [PMSF], and 50 mM sodium orthovanadate) and centrifuged at 17,000×g for 30 minutes at 4°C. Equal amounts of proteins were further separated using SDS-PAGE and then transferred to polyvinylidene fluoride (PVDF) membranes (Millipore, China). Membranes were blocked to avoid non-specific bindings in Tris-buffered saline-Tween (TBST) containing 5% fat-free milk for 2 hours and subsequently incubated with primary antibodies (shown in Table S6) at 4°C overnight. After three washes with TBST, membranes were incubated with the corresponding secondary antibody at a 1:10000 dilution at room temperature for 2 hours. Immunoblots were visualized by enhanced chemiluminescence (ECL kit, Santa Cruz Biotechnology) after washes. All the protein bands were scanned by Chem Imager 5500 V2.03 software and the integrated density values (IDVs) were calculated utilizing FluorChem 2.0 software.

**Cell immunofluorescence staining**

The Aβ_1-42_-incubated ECs were fixed by 4% paraformaldehyde for 20 minutes, and permeated in phosphate buffered saline (PBS) containing 0.2% Triton X-100 for 10 min. Next, ECs were blocked by 5% bovine serum album (BSA) in PBS for 2 hours at room temperature and incubated with primary antibodies (anti-ZO-1, 1:50, Life Technologies; anti-occludin, 1:50, Abcam; anti-claudin-5, 1:50, Life Technologies; anti-KHDRBS2, 1:50, Thermo fisher Scientific; anti-HEY2, 1:50, Proteintech) respectively at 4°C overnight. After three washes with PBS, ECs were incubated with fluorophore-conjugated secondary antibodies for 2 hours. DAPI were applied to observe cell nuclei. The staining was observed using confocal microscopy (Nikon C2, Japan) (the confocal microscopy parameters used were gain value of 2, gamma value of 1, and DAPI laser strength of 79%; Alexa Fluor is 68%).

**Immunohistofluorescence staining**

Mice were anesthetized with pentobarbital (40 mg/kg) and perfused with 30mL of PBS and 30mL of 4% paraformaldehyde from the left ventricle in turn. The mouse brain corpus striatum were excised and fixed in 4% paraformaldehyde overnight. Fixed brain tissue was embedded and cut into 10 μm slices. After antigen retrieval and washes, the slices were blocked in 8% donkey serum dissolved in 0.01 M PBS containing 0.5% Triton-X100 for 1h and incubated at 4°C overnight with anti-CD31(1:50, Abcam) and anti-ZO-1 (1:50, Thermo Scientific) dissolved in 1% donkey serum which was diluted from 8% donkey serum mentioned above using PBS. After washes, all sections were incubated at room temperature for 3 h with Cy3-conjugated IgG goat anti-rabbit (Jackson ImmunoResearch, Germany) and FITC-conjugated IgG goat anti-mouse (Jackson ImmunoResearch, Germany) diluted 1:300 in PBS containing 0.3% triton-X100. Nuclei were stained with DAPI for 20 min. Sections were observed using confocal microscopy (Nikon C2, Japan). We separated different channels and automatically generated pixel intensity for each channel. The ratio of ZO-1 (red) to CD31 (green) pixel intensity was calculated, and the average ratio of each image was used for statistical analysis.

**Fluorescence *in situ* hybridization (FISH)**

Aβ_1-42_-incubated ECs were fixed on slides in 4% formaldehyde (Sigma) for 15 min and then washed with PBS (containing 1% diethyl pyrocarbonate [DEPC], Dingguo, China) three times. After blocking with prehybridization buffer (3% BSA in 4 × saline-sodium citrate, SSC), slides were digested with PCR-grade proteinase K (Roche Diagnostics, Mannheim, Germany). The hybridization mix was prepared with ACTBP2 probe in the hybridization solution. Then the slides were washed with washing buffer and the sections were stained with anti-digoxin rhodamine conjugate (1:100, Exon Biotech Inc, Guangzhou, China) at 37°C for 1 h in the dark. Subsequently, the sections were stained with DAPI for nuclear staining. All images were observed with confocal microscope (Nikon C2, Japan).

**Chromatin immunoprecipitation assays**

ChIP assays were performed using a Simple-ChIP Enzymatic Chromatin IP Kit (Cell Signaling Technology, Danvers, MA, USA) following the manufacture’s description. Briefly, Aβ_1-42_-incubated ECs were crosslinked with 1% formaldehyde and collected in lysis buffer. Chromatin was then digested with Micrococcal Nuclease. Immunoprecipitates were incubated with 3μg corresponding antibody and anti-IgG and incubated with Protein G agarose beads at 4°C overnight with gentle shaking while 2% lysates were used as input reference. DNA crosslink was reversed with 5M NaCl and proteinase K and purified. Immunoprecipitation DNA was amplified by PCR using their specific primers. In each PCR reaction, the corresponding inputs were taken in parallel for PCR validation. Primers used for ChIP PCR are shown in Table S4 and Table S5.

**Reporter vector construction and luciferase reporter assays**

Human genomic DNA was used to amplify different promoter fragments, subcloned into pGL3-Basic-Luciferase vector (Promega) containing a firefly luciferase reporter gene and verified by DNA sequencing. Human full-length HEY2 was constructed in pEX3 (pGCMV/MCS/Neo) plasmid vector (GenePharma). HEK293T cells were co-transfected with the pGL3 vector of ZO-1, occludin and claudin-5 with full-length promoter regions (or deleted promoter regions) and pEX3-HEY2 (or pEX3 empty vector) using Lipofectamine 3000. Relative luciferase activity was calculated by normalizing to renilla luciferase activity.

**RNA immunoprecipitation (RIP) assays**

Whole cell lysate was incubated with human 5μg human anti-Ago2 antibody, or NC normal mouse IgG. Furthermore, purified RNA was extracted and applied to qRT-PCR to demonstrate the presence of the binding targets.

**RNA pull-down assays**

Biotin-labelled, full length ACTBP2, or antisense RNA was prepared with the Biotin RNA Labeling Mix (GenePharma, Shanghai, China) and transfected into ECs. Biotinylated RNAs were treated with RNase-free DNase I and purified. RNA-protein complexes were isolated by streptavidin agarose beads (Invitrogen, Shanghai, China) and washed three times. The retrieved proteins were detected using a standard western blotting technique with GAPDH as the control.

**Chromatin Isolation by RNA Purification assays**

Cells were fixed by 4% paraformaldehyde. Then the crosslinked chromatin was sonicated and hybridized using antisense DNA oligonucleotide probes specific for full-length ACTBP2 (designed on https://www.biosearchtech.com and showed in Table S7) ans LacZ RNA. All probes were biotinylated at the end (RiboBio, Guangzhou, China). The hybridized ACTBP2-chromatin was captured by streptavidin-labeled beads, and RNA and DNA were subsequently purified. ACTBP2-binding chromatin was detected by real-time PCR. The data was normalized to GAPDH (negative control).
