# Supplementary figures and images for: Pseudogene ACTBP2 increases blood–brain barrier permeability by promoting KHDRBS2 transcription through recruitment of KMT2D/WDR5 in Aβ1–42 microenvironment
Source: Cell Death Discov. 2021 Jun 14;7:142. doi: 10.1038/s41420-021-00531-y (PMC8203645; doi:10.1038/s41420-021-00531-y)

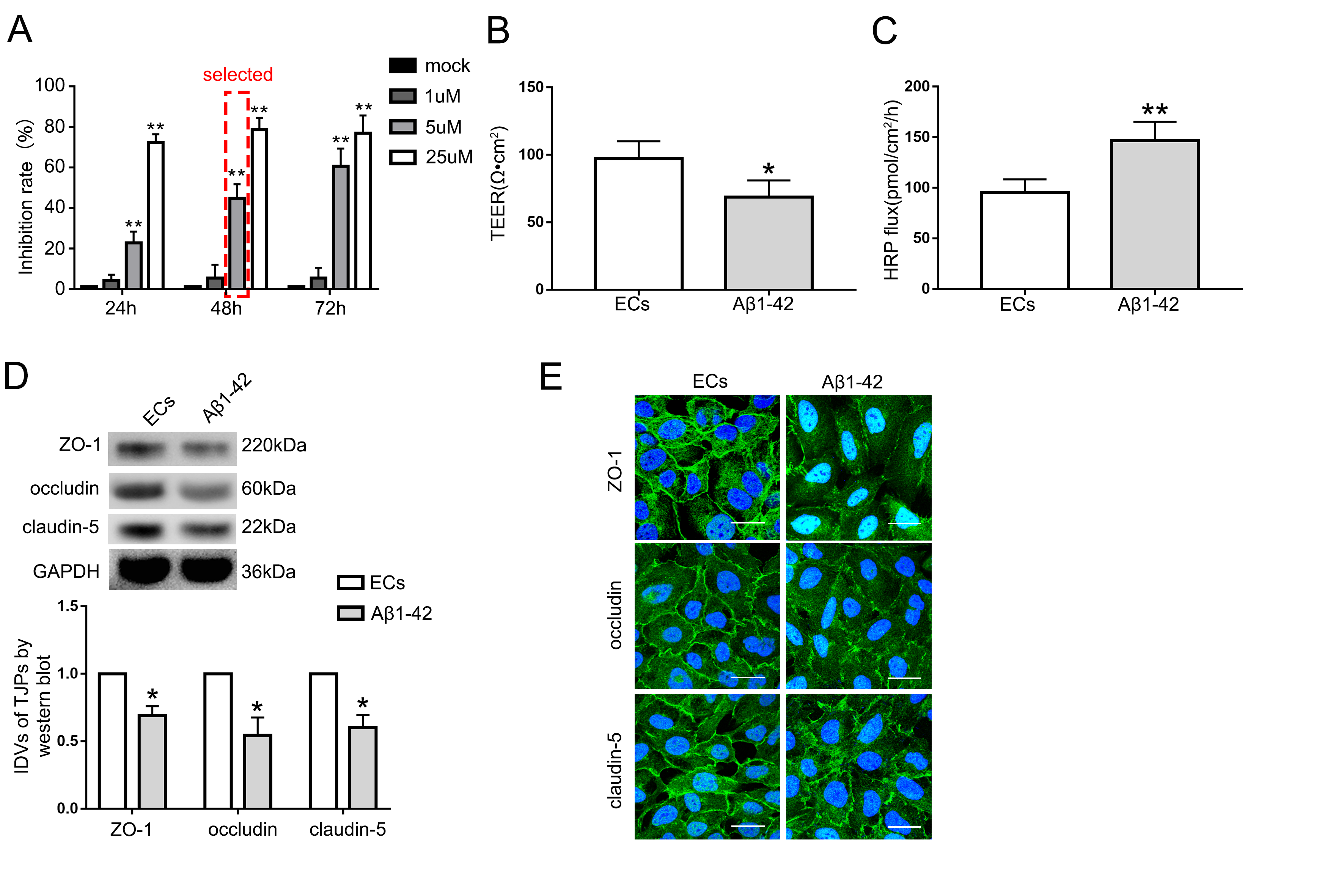

Supplement: Supplementary file 2 — Figure S1 [file 41420_2021_531_MOESM2_ESM.tif]

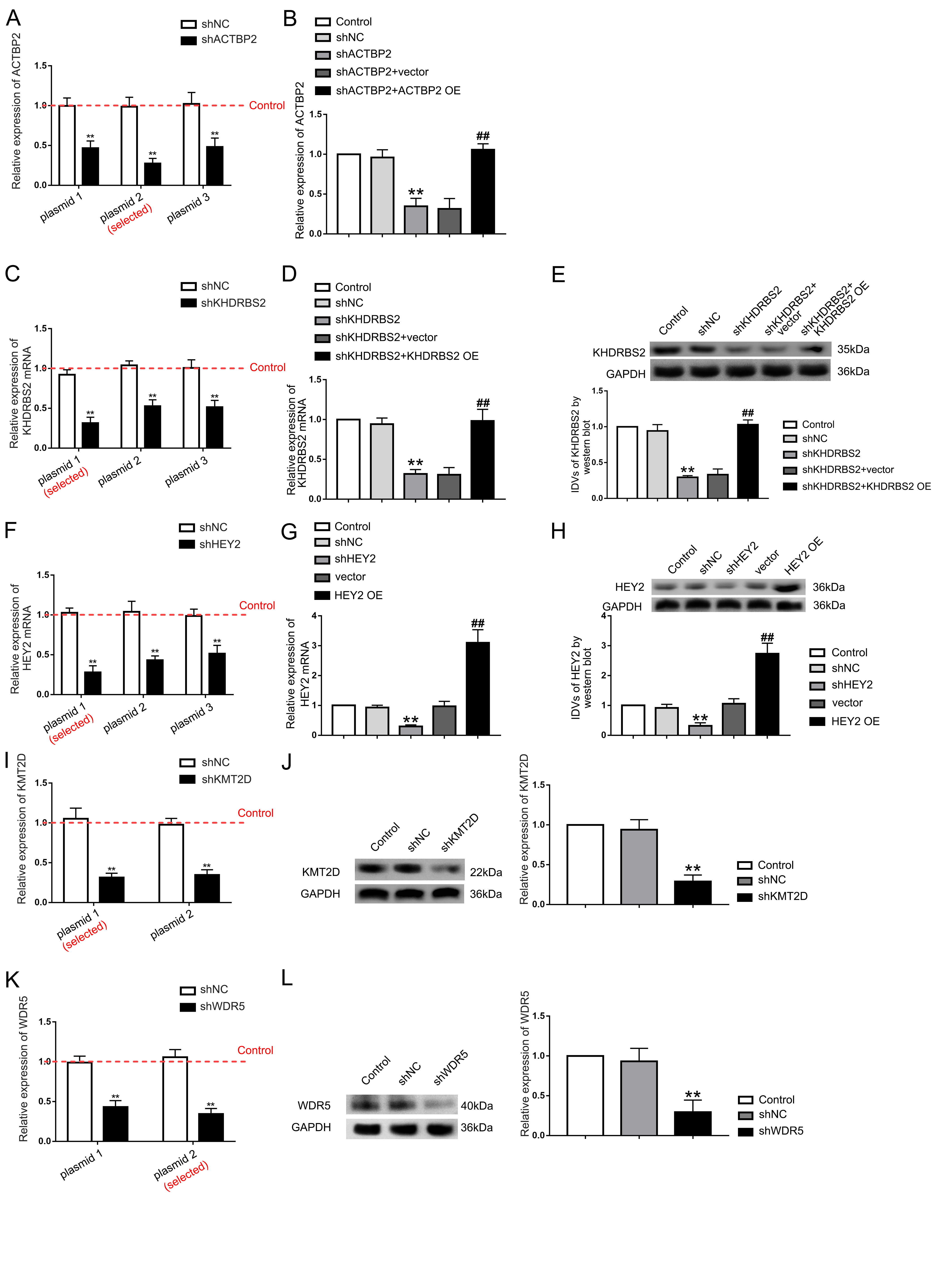

Supplement: Supplementary file 3 — Figure S2 [file 41420_2021_531_MOESM3_ESM.tif]

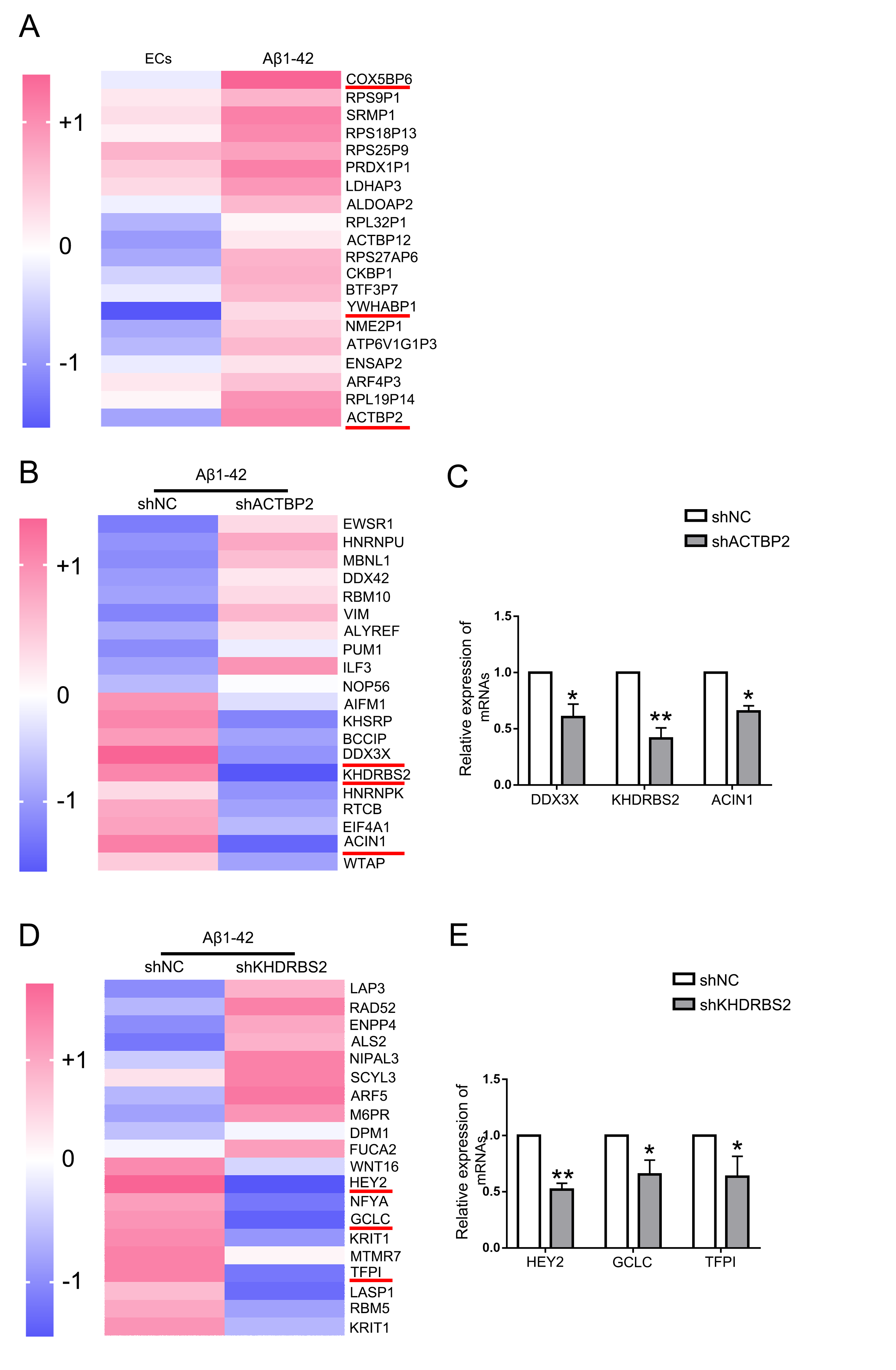

Supplement: Supplementary file 4 — Figure S3 [file 41420_2021_531_MOESM4_ESM.tif]

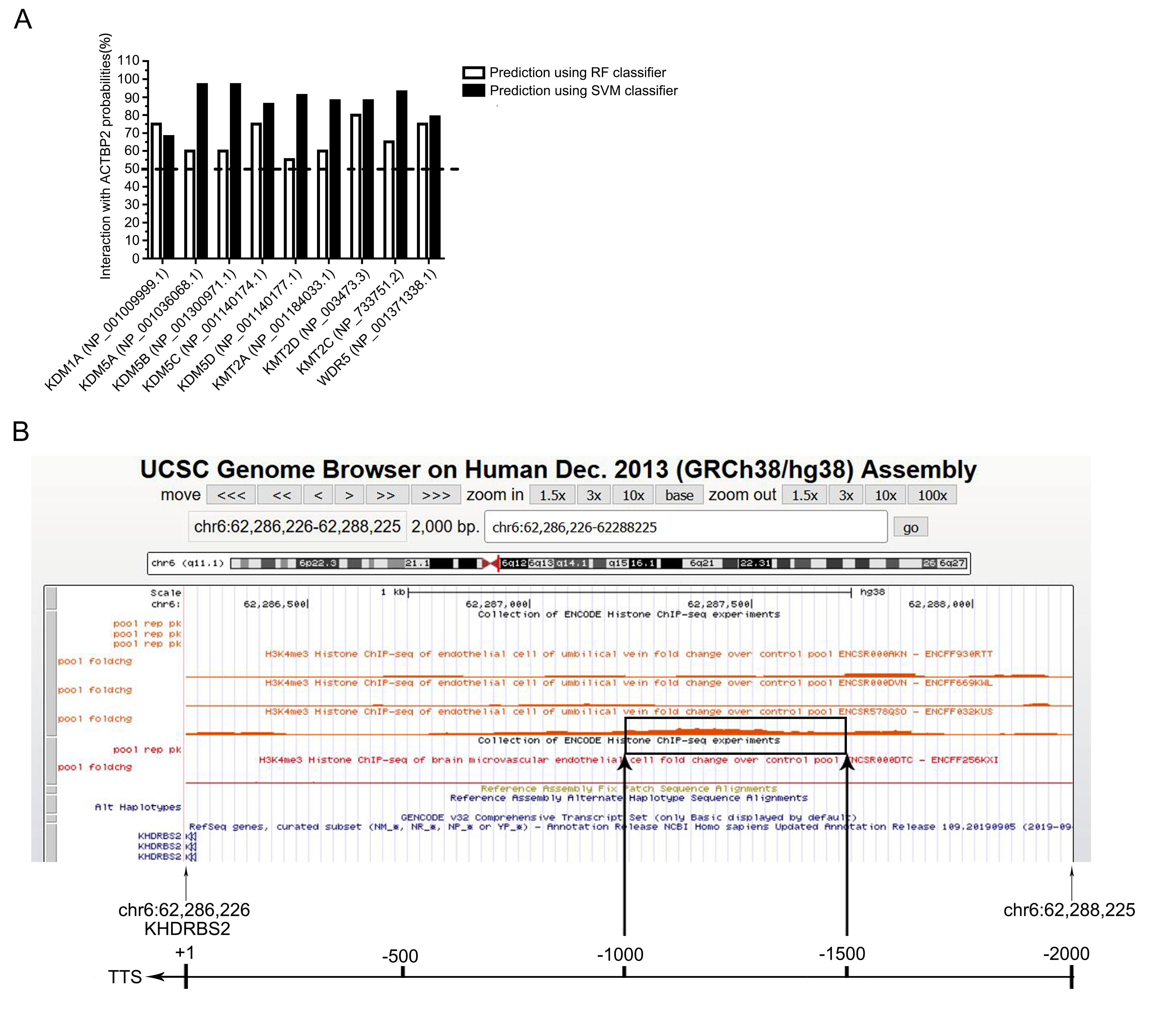

Supplement: Supplementary file 5 — Figure S4 [file 41420_2021_531_MOESM5_ESM.tif]

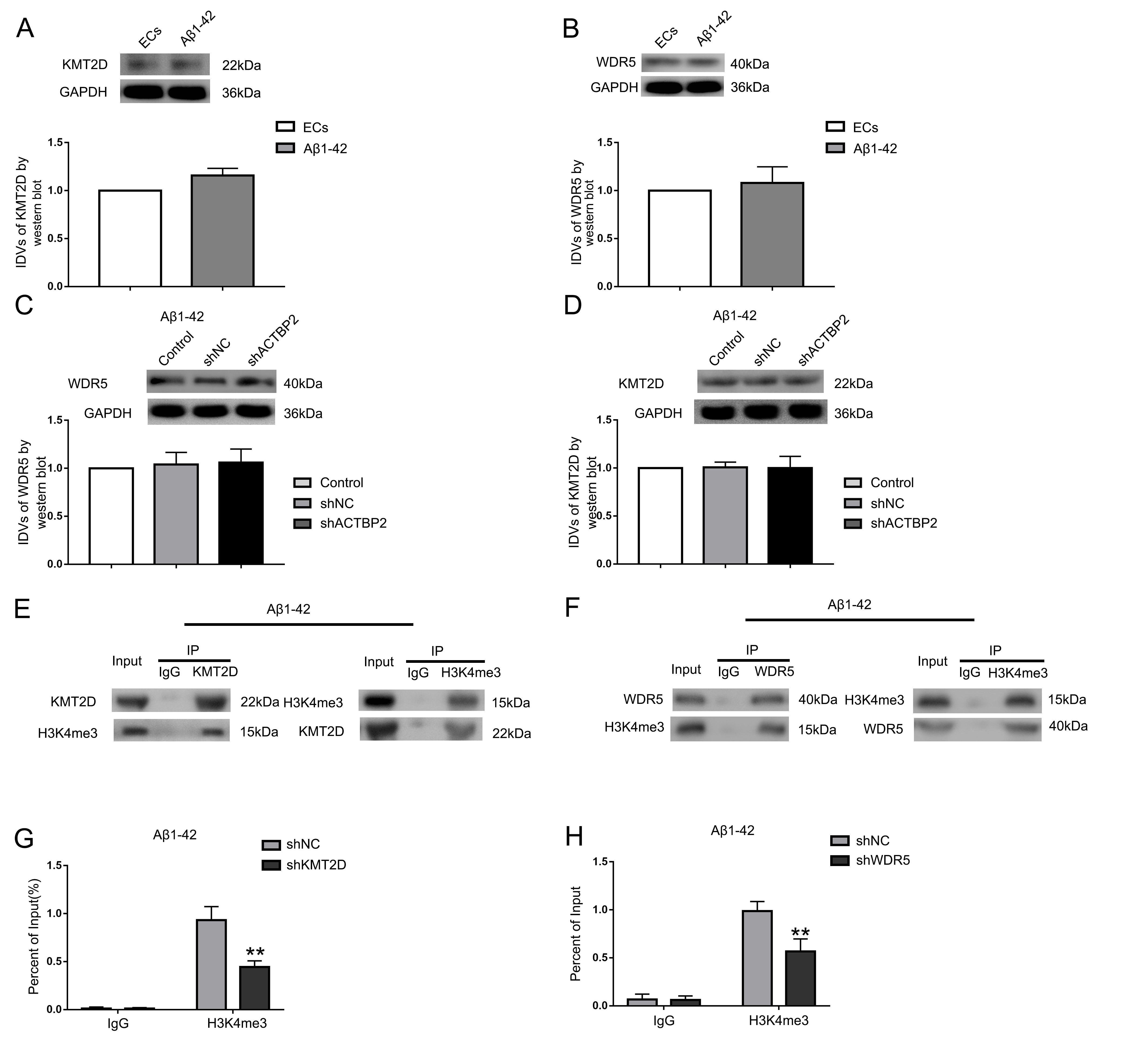

Supplement: Supplementary file 6 — Figure S5 [file 41420_2021_531_MOESM6_ESM.tif]

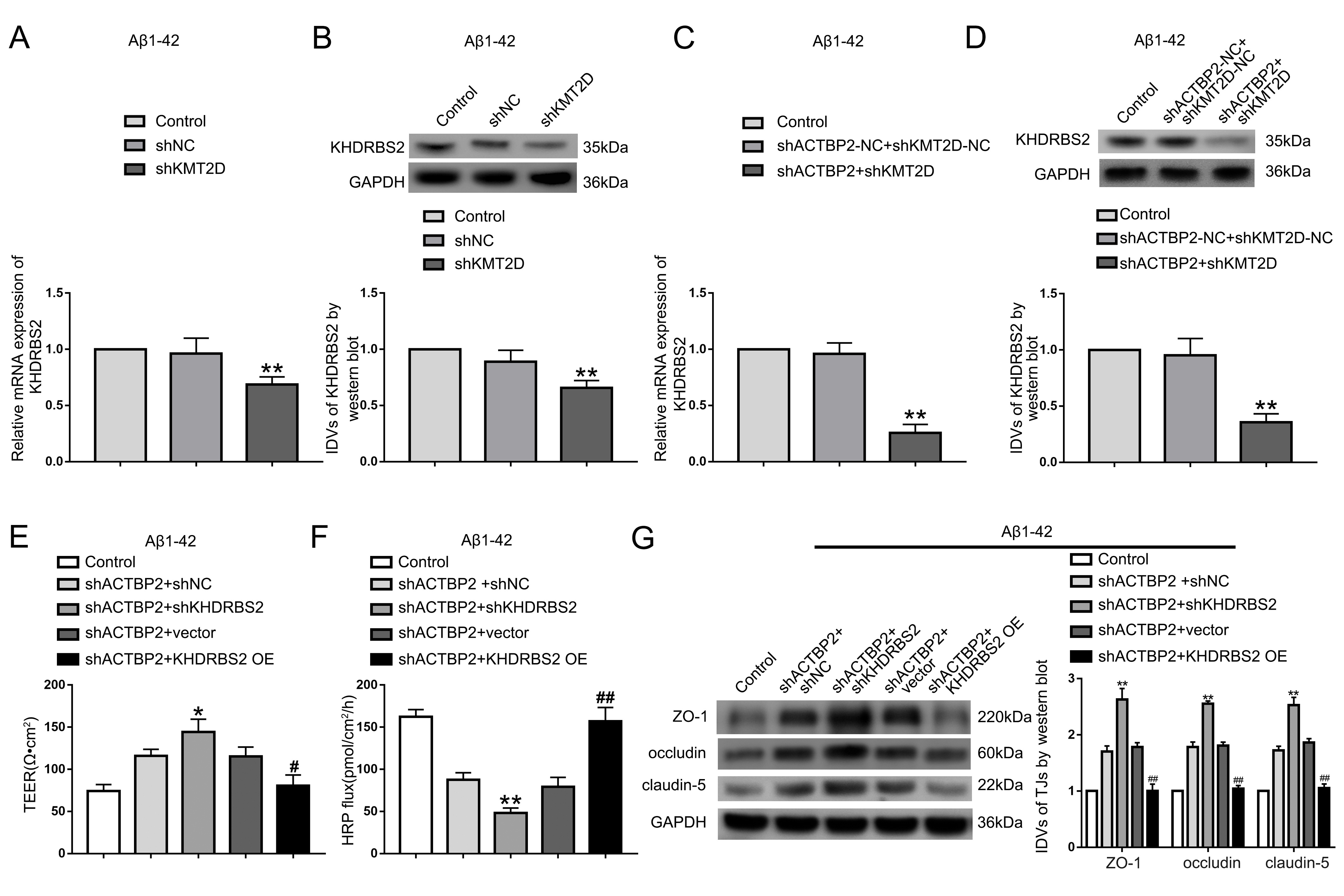

Supplement: Supplementary file 7 — Figure S6 [file 41420_2021_531_MOESM7_ESM.tif]

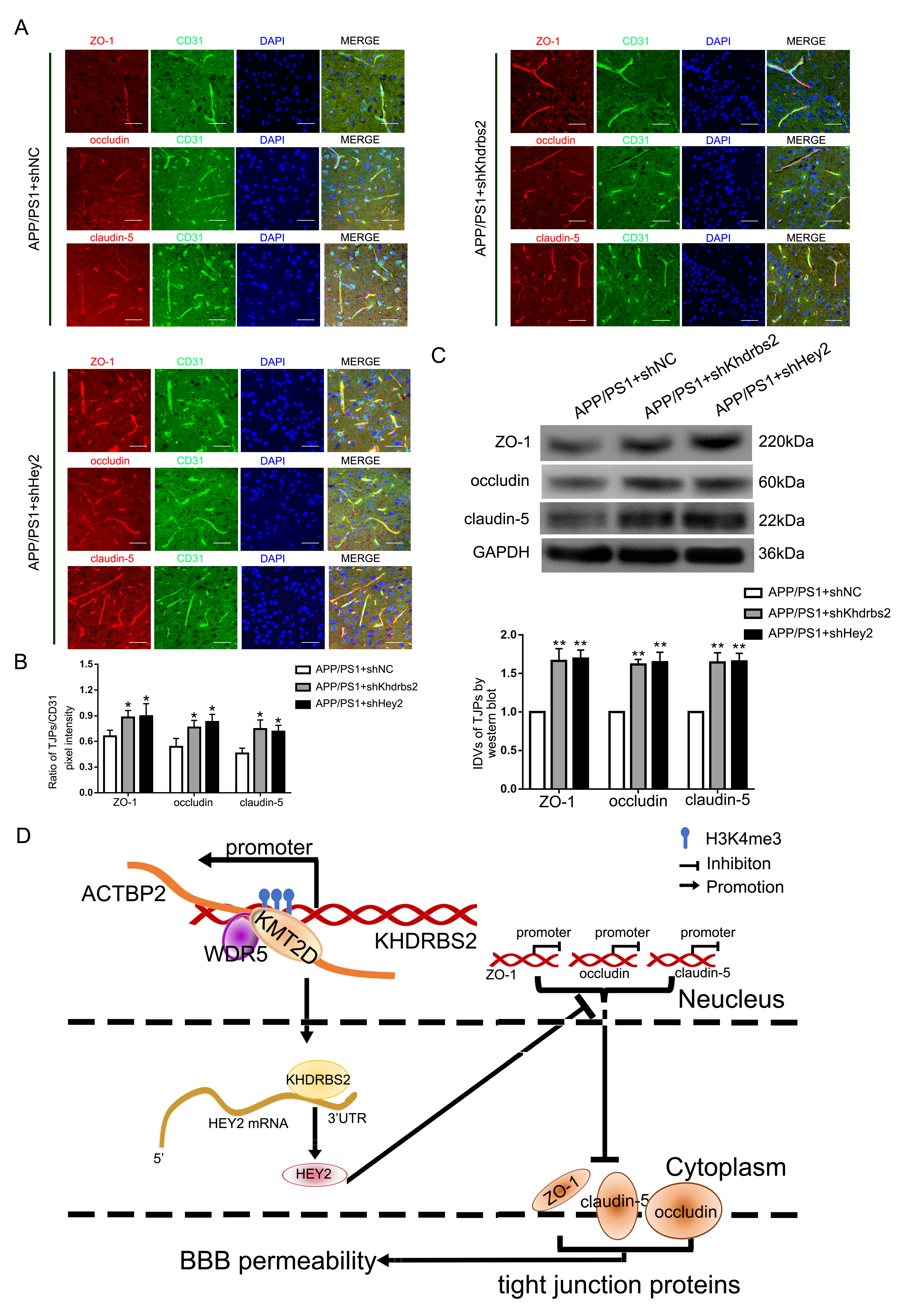

Supplement: Supplementary file 8 — Figure S7 [file 41420_2021_531_MOESM8_ESM.tif]

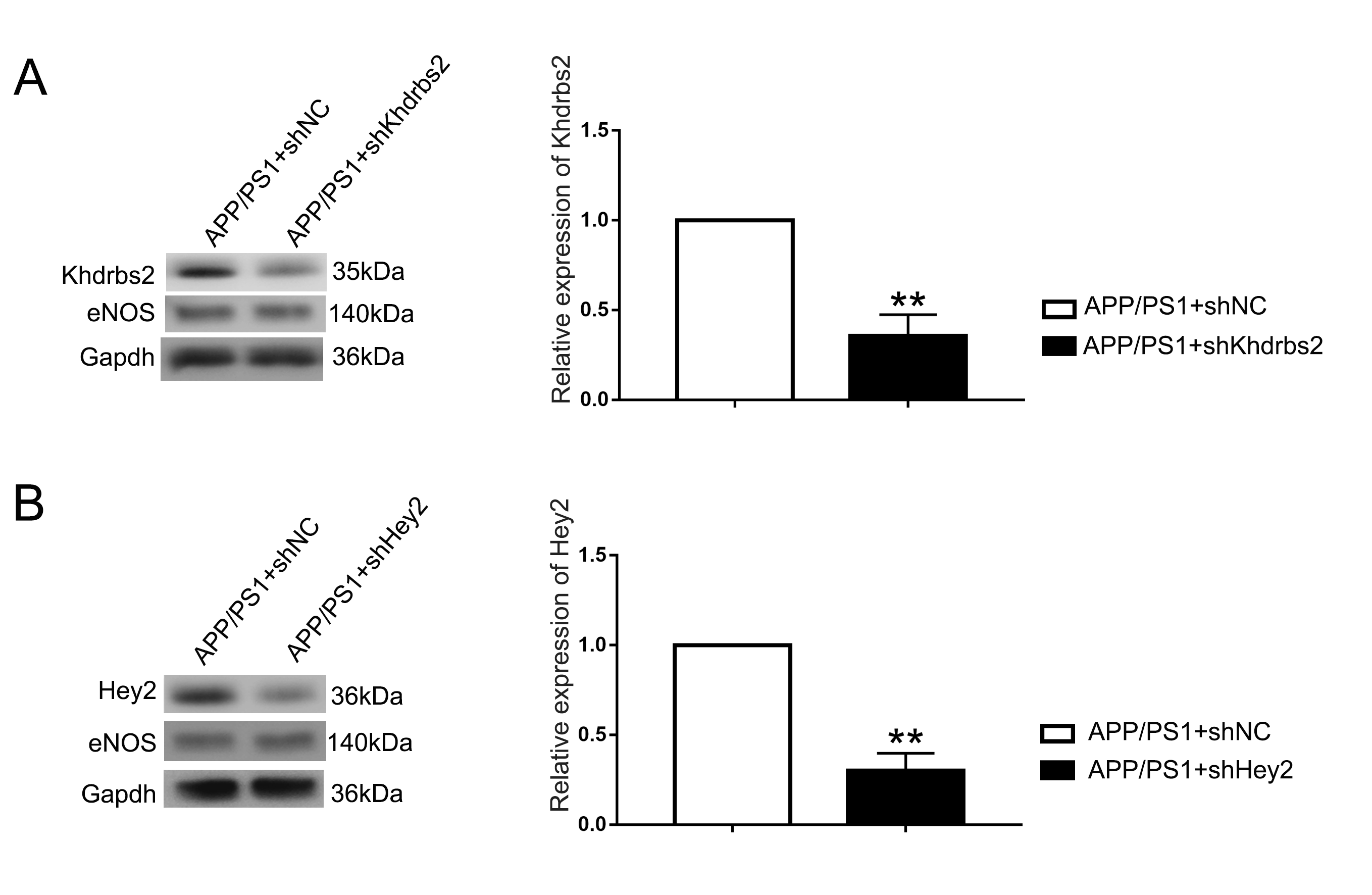

Supplement: Supplementary file 9 — Figure S8 [file 41420_2021_531_MOESM9_ESM.tif]
