# Supplementary material for: Pseudogene ACTBP2 increases blood–brain barrier permeability by promoting KHDRBS2 transcription through recruitment of KMT2D/WDR5 in Aβ1–42 microenvironment
Source: Cell Death Discov. 2021 Jun 14;7:142. doi: 10.1038/s41420-021-00531-y (PMC8203645; doi:10.1038/s41420-021-00531-y)
Supplement: Supplementary file 10 — Supplemental Tables [file 41420_2021_531_MOESM10_ESM.pdf]

Table S1. Primers used for RT-qPCR

Primer or Probe	Gene	Sequence (5'->3') or Assay ID
Primer	ACTBP2	F: TGGTGGGCATGGGTCAGAAGG
		R: AGAGGCGTACAGGGACAGCATAG
	KHDRBS2	F: ATCCTGGGCAAAGGGTCCAT
		R: CGGGTGGAGCAAACACTTC
	HEY2	F: CCCACGGCTCTGCTCTCCTC
		R: ACTGCTGCTGCTGCGTTTGG
	ZO-1	F: AGTGCTGGCTTGGTCTGTTTGC
		R: GACGCTGGGTGATAGGGATTTGTG
	occludin	F: AACTTCGCCTGTGGATGACTTCAG
		R: GACCTTCCTGCTCTTCCCTTTGC
	Claudin-5	F: CGCCTTCCTGGACCACAACATC
		R: AGAGCCAGCACCGAGTCGTAC
	GAPDH	F: CGGATTTGGTCGTATTGGG
		R: CTGGAAGATGGTGATGGGATT

Table S2. Human shRNA target sequences

Gene		Sequence(5'->3')
ACTBP2	Sence	CACCGCACCACACCTTCTACAATGACGAATCATTGTAGAAGGTGTGGTGC
(human)	Antisence	AAAAGCACCACACCTTCTACAATGATTCGTCATTGTAGAAGGTGTGGTGC
KHDRBS2	Sence	CACCGGGAAATTGCTTGGACCAAGACGAATCTTGGTCCAAGCAATTTCCC
(human)	Antisence	AAAAGGGAAATTGCTTGGACCAAGATTCGTCTTGGTCCAAGCAATTTCCC
HEY2	Sence	CACCGCGTCGGGATCGGATAAATAACGAATTATTTATCCGATCCCGACGC
(human)	Antisence	AAAAGCGTCGGGATCGGATAAATAATTCGTTATTTATCCGATCCCGACGC
KMT2D	Sence	CACCGCTGTCCATGGAAACCTAAACCGAAGTTTAGGTTTCCATGGACAGC
(human)	Antisence	AAAAGCTGTCCATGGAAACCTAAACTTCGGTTTAGGTTTCCATGGACAGC
WDR5	Sence	CACCGCACACTAGCACTTTCCTACTCGAAAGTAGGAAAGTGCTAGTGTGC
(human)	Antisence	AAAAGCACACTAGCACTTTCCTACTTTCGAGTAGGAAAGTGCTAGTGTGC
NC	Sence	CACCGTTCTCCGAACGTGTCACGTCAAGAGATTACGTGACACGTTCGGAGAATTTTTG
	Antisence	GATCCAAAAAAGTTCTCCGAACGTGTCACGTAATCTCTTGACGTGACACGTTCGGAGAAC

Table S3. Mouse shRNA target sequences

Gene		Sequence(5'->3')
Khdrbs2	Sence	CACCGGGAGTTGTCTTACTTGAATGCGAACATTCAAGTAAGACAACTCC
(mouse)	Antisence	AAAAGGGAGTTGTCTTACTTGAATGTTCGCATTCAAGTAAGACAACTCCC
Hey2	Sence	CACCGCAAATGACAGTGGATCATTTCGAAAAATGATCCACTGTCATTTGC
(mouse)	Antisence	AAAAGCAAATGACAGTGGATCATTTTTCGAAATGATCCACTGTCATTTGC

Table S4. Primers used for confirming the interaction between HEY2 and the promotor region of ZO-1, occludin and claudin-5

Gene	Binding site or Control	Sequence (5'->3')	Product size (bp)	Annealing temperature (°C)
KHDRBS2	PCR1	F:TCCTACTGCGTCCCTGAGAT<br>R:CCTCCGCCAACCACTCATT	500	55.2
	PCR2	F:TCCTAGCGATTGAGGCATGCA<br>R:AGCTGGTATTTCCGATGACCTACATC	500	56.1
	PCR3	F:TTTCAGTATCCAATAAACATTTGTTGAATG<br>R:AACAATCTTTCCCCAAGTCTTG	500	51.4
	PCR4	F:AGAATAAAATCCAAATAATTTTCATTCATCAAATC<br>R:AGAGTCTAGGGACAGTACTTAAAATT	500	52.4
	PCR5	F:ATCATGGAAAAGATGGCTGTG<br>R:AAGTGTAATAGGGGACCATAAAGTATT	500	50.6
ZO-1	PCR1	F: CCAGTGTACTCCCTTTTATGGA<br>R: CCTGGAAGCTGAGGGACCTA	110	51
	PCR2	F: AGCCGTCAACATTGTGGGAA<br>R: TCCAAAGTCTCTTTCCCGCC	218	55.6
	PCR3	F:AACGAGAGCAACGCTTCTGA<br>R:GACAAGCCACGCACATCATG	228	56.6
occludin	PCR1	F: TCCCAGCTACTCAAGAGGCT<br>R: GCATGATCACTTGGGCTCCT	222	53
	PCR2	F: CCCAGGAGTCTTTCGTTGGA<br>R: AGGTCCAGAGGGGACTGTTT	162	55.7
Claudin-5	PCR1	F: AGGATCCCTTGAGACCAGGA<br>R: TATGCACCACCACCCACCTA	101	51
	PCR2	F: GGAGATGTCCAGGAGGGTCT<br>R: GTGTGGGGAGGTTCTGAAGG	209	58.3
	PCR3	F: GAAACAAAGGGGCCGAGAGA<br>R: TCGAAAGGTCCACTGGTGTG	140	57.4

Table S5. The primers for confirming the existence of H3K4me3 modification in KHDRBS2 promotor region.

Gene	Binding site or Control	Sequence (5'->3')	Product size (bp)	Annealing temperature (°C)
KHDRBS2	PCR1	F:TCCTACTGCGTCCCTGAGAT<br>R:CCTCCGCCAACCACTCATT	500	55.2
	PCR2	F:TCCTAGCGATTGAGGCATGCA<br>R:AGCTGGTATTTCCGATGACCTACATC	500	56.1
	PCR3	F:TTTCAGTATCCAATAAACATTTGTTGAATG<br>R:AACAATCTTTCCCCAAGTCTTG	500	51.4
	PCR4	F:AGAATAAAATCCAAATAATTTTCATTCATCAAATC<br>R:AGAGTCTAGGGACAGTACTTAAAATT	500	52.4
	PCR5	F:ATCATGGAAAAGATGGCTGTG<br>R:AAGTGTAATAGGGGACCATAAAGTATT	500	50.6

Table S6. Antibodies for western blot

Gene	Antibody
KHDRBS2	1:500, Thermo fisher Scientific, Waltham, MA, USA
HEY2	1:500, Proteintech, Wuhan, CHN
KMT2D	1:500, Thermo fisher Scientific, Waltham, MA, USA
WDR5	1:500, Thermo fisher Scientific, Waltham, MA, USA
ZO-1	1:500, Life Technologies Corporation, USA
occludin	1:600, Abcam, Cambridge, UK
claudin-5	1:300, Life Technologies Corporation, Paisley, UK
eNOS	1:200, Cell Signaling Technology, CHN

Table S7. The probes of ACTBP2 and KHDRBS2 in Chromatin Isolation by RNA Purification

Probe name	Probe sequence (5'->3')	Position of first nucleotide of probe
ACTBP2 asDNA#1	TGACAATGCCGTGCTCAATG	294
ACTBP2 asDNA#2	TCGTAGATGGGCACAGTGTG	565
ACTBP2 asDNA#3	TCGTTGCCAATGGTGATGAC	818
ACTBP2 asDNA#4	AATCCACACAGAGTACTTGC	1083
ACTBP2 asDNA#5	GGACTTCCTGTAACAATGCA	1474
ACTBP2 asDNA#6	TTCATACATCTCAAGCTGGG	1691

Probe name	Probe sequence (5'->3')	Position of first nucleotide of probe
ACTBP2	TGAAGACTGCATGTGATCGG	471
KHDRBS2 promoter-1	AGGTAGATGGGACTTCACCT	87
KHDRBS2 promoter-2	GCGGCTCGAAGAAATATTGT	385
KHDRBS2 promoter-3	GGGCCAACTTTGCTACAAAT	364
KHDRBS2 promoter-4	GGTCAAGAATGAACCAAGGA	330
KHDRBS2 promoter-5	GTCCAGCAACAACAACTACT	30
