# Supplementary material for: Pseudogene ACTBP2 increases blood–brain barrier permeability by promoting KHDRBS2 transcription through recruitment of KMT2D/WDR5 in Aβ1–42 microenvironment
Source: Cell Death Discov. 2021 Jun 14;7:142. doi: 10.1038/s41420-021-00531-y (PMC8203645; doi:10.1038/s41420-021-00531-y)
Supplement: Supplementary file 11 — Spplemental Figure legends [file 41420_2021_531_MOESM11_ESM.docx]

**Figure S1 The effects of Aβ_1-42_ on BBB permeability.** (A) The effects of Aβ_1-42_ on ECs growth *in vitro*. ECs were incubated with Aβ_1-42_ for different time and growth inhibitory effects were assayed by CCK-8. Data are presented as mean ± SD (n = 3, each). ***^**^****P* < 0.01 *versus* mock group. (B and C) Effects of Aβ_1-42_ on TEER values (B) and HRP flux (C). (D) Effects of Aβ_1-42_ on ZO-1, occludin and claudin-5 expression levels in ECs. Data are presented as mean ± SD (n = 3, each). ***^*^****P* < 0.05 *versus* ECs group. ***^**^****P* < 0.01 *versus* ECs group. (E) Effects of Aβ_1-42_ on ZO-1, occludin and claudin-5 expression levels and distribution determined by immunofluorescence staining. ZO-1, occludin and claudin-5 (green) were labeled with secondary antibody against anti-ZO-1, anti-occludin and anti-claudin-5 antibody, respectively, and nuclei (blue) were labeled with DAPI. Scale bar represents 30 μm.

**Figure S2 Transfection efficiency of Aβ_1-42_-incubated ECs.** (A) The transfection efficiency of ACTBP2 knockdown in Aβ_1-42_-incubated ECs. Data are presented as mean ± SD (n = 3). ***^**^****P* < 0.01 *versus* shNC group. (B) Expression of ACTBP2 by qRT-PCR upon re-expressing or depleting ACTBP2. Data are presented as mean ± SD (n = 3). ***^**^****P* < 0.01 *versus* shNC group, ***^##^****P* < 0.01 *versus* shACTBP2+vector group. (C) The transfection efficiency of KHDRBS2 knockdown. Data are presented as mean ± SD (n = 3). ***^**^****P* < 0.01 *versus* shNC group. (D and E) Expression of KHDRBS2 by qRT-PCR (D) and western blot (E) upon shKHDRBS2 or re-expressing KHDRBS2 in shKHDRBS2 cells. Data are presented as mean±SD (n = 3, each). ***^**^****P* < 0.01 *versus* shNC group, ***^##^****P* < 0.01 *versus* shKHDRBS2+vector group. (F) Expression of HEY2 when depleting HEY2 and overexpressing HEY2. Data are presented as mean ± SD (n = 3). ***^**^****P* < 0.01 *versus* shNC group. (G and H) Expression of HEY2 by qRT-PCR (G) and western blot (H) upon silencing or over-expressing HEY2. Data are presented as mean ± SD (n = 3, each). ***^**^****P* < 0.01 *versus* shNC group. ^##^*P* < 0.01 *versus* vector group. (I and J) The transfection efficiency of KMT2D knockdown in Aβ_1-42_-incubated ECs by qRT-PCR (I) and western blot (J). (K and L) Expression of WDR5 by qRT-PCR (K) and western blot (L). Data are presented as mean ± SD (n = 3, each). ***^**^****P* < 0.01 *versus* shNC group.

**Figure S3 Selection of ACTBP2, KHDRBS2 and HEY2.** (A) Pseudogenes microarray analysis was performed in Aβ_1-42_-incubated ECs. Red indicates high relative expression and blue indicates low relative expression. (B) RNA microarray analysis was performed in Aβ_1-42_-incubated ECs treated with shACTBP2. Red indicates high relative expression and blue indicates low relative expression. (C) Relative expression of DDX3X, KHDRBS2 and ACIN1 in Aβ_1-42_-incubated ECs by qRT-PCR. Data are presented as mean ± SD (n = 3), ***^*^****P* < 0.05 *versus* shNC group, ***^**^****P* < 0.01 *versus* shNC group. (D) RNA microarray analysis was performed in Aβ_1-42_-incubated ECs treated with shKHDRBS2. Red indicates high relative expression and blue indicates low relative expression. (E) Relative expression of HEY2, GCLC and TFPI in Aβ_1-42_-incubated ECs by qRT-PCR. Data are presented as mean ± SD (n = 3), ***^*^****P* < 0.05 *versus* shNC group, ***^**^****P* < 0.01 *versus* shNC group.

**Figure S4 Database prediction related to Figure 3.** (A) RNA-protein interaction prediction (RPISeq) analysis was used to predict the interaction between ACTBP2 and H3K4 (de)methyltransferases (http://pridb.gdcb.iastate.edu/RPISeq/), predictions with probabilities > 0.5 were considered positive. (B) Existence of H3K4me3 in KHDRBS2 promoter region 1000-1500 bp upstream of the transcription start site (TSS) predicted by Encyclopedia of DNA Elements (ENCODE) databse (https://www.encodeproject.org/).

**Figure S5 Related to Figure 3.** (A and B) Endogenous expression of KMT2D (A) and WDR5 (B) in Aβ_1-42_-incubated ECs. (C and D) Effects of ACTBP2 knockdown on WDR5 (C) and KMT2D (D) expression by Western blot. Data are presented as mean ± SD (n = 3, each). (E) Co-immunoprecipitation detected the interaction of KMT2D and H3K4me3 in Aβ_1-42_-incubated ECs. The specific immunoprecipitation of KMT2D and H3K4me3 was confirmed by Western blot. (F) Co-immunoprecipitation confirmed that WDR5 interacted with H3K4me3. (G and H) H3K4me3 levels in the KHDRBS2 promoter region from 1000 to 1500 bp upstream of the transcription start site (TSS) after KMT2D knockdown (G) and WDR5 knockdown (H) by qRT-PCR. Data are presented as the mean ± SD (n = 3, each group). ***^**^****P* < 0.01 *versus* shNC group.

**Figure S6 Related to Figure 3. The effects of ACTBP2 on BBB permeability were mediated by KHDRBS2 in Aβ_1-42_ microenvironment.** (A) Effects of KMT2D knockdown on KHDRBS2 mRNA expression level by qRT-PCR. (B) Effects of KMT2D knockdown on KHDRBS2 protein expression level by western blot. Data are presented as mean ± SD (n = 3, each). ***^**^****P* < 0.01 *versus* shNC group. (C) Effects of co-knockdown of ACTBP2 and KMT2D on KHDRBS2 mRNA expression level. (D) Effects of co-knockdown of ACTBP2 and KMT2D on KHDRBS2 protein expression level. Data are presented as mean ± SD (n = 3, each). ***^**^****P* < 0.01 *versus* shACTBP2+shNC group. (E and F) Effects of ACTBP2 knockdown and KHDRBS2 on TEER values (E) and HRP flux (F) in Aβ_1-42_ microenvironment. (G) Effects of ACTBP2 knockdown and KHDRBS2 on ZO-1, occludin, and claudin-5 expression levels in Aβ_1-42_-incubated ECs determined by western blot. Data are presented as mean ± SD (n = 3, each). ***^*^****P* < 0.05 *versus* shACTBP2+shNC group. ***^**^****P* < 0.01 *versus* shACTBP2+shNC group. ***^#^****P* < 0.05 *versus* shACTBP2+vector group. ***^##^****P* < 0.01 *versus* shACTBP2+vector group.

**Figure S7** (A) The predicted interaction of RBP-KHDRBS2 and HEY2 mRNA according to a bioinformatics database (starBase). (B-D) The potential binding sites of HEY2 in the 1,000bp upstream promoter region of ZO-1 (B), occludin (C) and claudin-5 (D) were found by JASPAR.

**Figure S8 Transfection efficiency of AD transgenic mice brain microvessels.** (A) The transfection efficiency of Khdrbs2 knockdown in mice brain microvessels by western blot. (B) The transfection efficiency of Hey2 knockdown in mice brain microvessels by western blot. Data are presented as the mean ± SD (n = 3, each group). ***^**^****P* < 0.01 *versus* APP/PS1+shNC group.
